# Emerging Decoupled Water Electrolysis for Green Hydrogen Production: Current Progress and Future Roadmap

**DOI:** 10.1002/cssc.71166

**Published:** 2026-10-24

**Authors:** Anindita Chakraborty, Bokinala Moses Abraham, Joydeep Dutta, Ujjwal Pal

**Affiliations:** ^1^ Department of Energy and Environmental Engineering CSIR‐Indian Institute of Chemical Technology Hyderabad India; ^2^ School of Chemistry University of Leeds Leeds UK; ^3^ Functional Nanomaterials Group Department of Applied Physics KTH Royal Institute of Technology Stockholm Sweden

**Keywords:** decoupled water electrolyzer, DFT/AIMD, green hydrogen, membranes, redox mediators

## Abstract

Decoupled water electrolysis (DWE) offers a unique strategy for green hydrogen production by separating the hydrogen and oxygen evolution reactions either in time or space, with the potential for integration to intermittent renewable energy sources. This review highlights the critical assessment of DWE, including the different components used and the operational strategies to accelerate the hydrogen economy. Special emphasis is placed on redox mediator engineering, catalyst innovation, membranes, and next‐generation cell architectures to overcome current limitations associated with conventional electrolyzers including efficiency, stability, and cost. The in silico approaches, including density functional theory and molecular dynamics‐driven parameter optimization can accelerate DWE by bridging the gap between lab‐scale technology development and industrial scale‐up, thereby facilitating the rise of DWE as one of the next‐generation technologies for green hydrogen production. A visionary roadmap is proposed, showing the pathway from proof‐of‐concept laboratory studies toward market deployment, with the aim of recognizing DWE as a cornerstone technology for a sustainable, low‐carbon hydrogen future. The review is conceptualized based on literature survey and identified research gaps.

## Graphical abstract

Decoupled water electrolysis redefines green hydrogen production by separating hydrogen and oxygen evolution in time or space, enabling safer, more flexible integration with intermittent renewables. This review critically examines redox mediators, catalysts, membranes, advanced cell architectures, and computational design, while charting a visionary pathway from laboratory innovation to scalable, market‐ready hydrogen technologies.

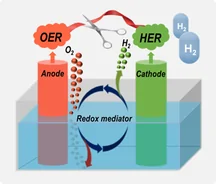

## 1 Introduction

The increasing necessity to reduce carbon emissions in the economy has driven the quest for clean energy production from sustainable sources and the adoption of eco‐friendly fuels. Green hydrogen is regarded as a promising alternative to minimize the reliance on natural fuel sources owing to its high energy density and potential use as a raw material for chemical synthesis or as an energy carrier for fuel cells and combustion engines, thus mitigating the carbon emanation in the ecosystem. Today, most of the industrial hydrogen is generated through steam methane reforming process, and this to 95% of global production contributes roughly to 3% of worldwide CO_2_ emissions [1, 2]. Hydrogen generation by splitting water using various techniques, including chemical oxidation, photocatalysis [3, 4], photoelectrochemical processes [5, 6], thermochemical reactions [7], and electrolysis [8, 9], is more sustainable. Recently, the high demand for decarbonization strategies has led to swift commercialization of cost‐effective renewable energy systems, leading to the intensified interest in hydrogen production via water electrolysis using renewable electricity supply, enabling the production of clean hydrogen with almost zero carbon emissions.

Four technologies of water electrolysis such as proton exchange membrane (PEM) electrolyzer, anion exchange membrane (AEM) electrolyzer, solid oxide electrolyzer (SOE), and alkaline water electrolyzer (AWE) have been commercially scaled [10, 11, 12, 13, 14, 15]. Each technology operates with distinct electrolytes and follows different mechanisms to facilitate the electrochemical splitting of water. Because hydrogen evolution reaction (HER) and oxygen evolution reaction (OER) occur concurrently, conventional electrolyzers depend on specialized membranes to restrict the gas crossover and maintain efficient ion transport. PEM electrolyzers are operated in an acidic environment, where protons migrate through the membrane, whereas AEM electrolyzers work in an alkaline medium, where hydroxide ions move across the membrane. As the membrane is one of the critical components in conventional electrolyzers, membrane durability is a significant concern in both AEM and PEM [16]. During prolonged electrolysis, the membranes were subjected to chemical and mechanical degradation, which severely impacts the electrolyzer's efficiency and lifespan. Chemical degradation in membrane occurs due to continuous exposure to harsh electrochemical environments, oxidative degradation, and interactions with contaminants or byproducts in the electrolyte [17]. Whereas, mechanical degradation occurs from operational stresses such as pressure fluctuations, swelling and contraction, and physical strain during prolonged use leading to thinning, cracking, or even complete membrane failure, necessitating periodic membrane replacement [18]. Frequent membrane replacement not only increases maintenance efforts but also increases operational costs due to downtime, rendering membrane durability a key challenge for the economic feasibility of PEM and AEM electrolyzers. Unlike PEM and AEM electrolyzers, SOE relies on dense ceramic oxide‐ion‐conducting electrolytes, such as yttria‐stabilized zirconia, scandia‐stabilized zirconia, or gadolinium‐doped ceria (GDC), to transport oxide ions generated from steam reduction from cathode to anode where OER occurs. SOE has the potential to function at higher energy efficiency through waste heat recovery, but it is in the early stages of development [19]. The AWE uses a liquid electrolyte and a porous diaphragm to prevent gas crossover, but some gas mixing can still restrict the applications of AWE. In all conventional methods of water electrolysis, hydrogen and oxygen generation occur simultaneously in a single electrochemical cell, which can potentially lead to accumulation of the produced gases in a closed chamber that can be explosive, posing a major safety risk. Diffusion of gases also reduces the purity of the produced hydrogen, making it less suitable for high‐purity applications such as fuel cells and industrial hydrogen use without subsequent gas purification steps. Another crucial challenge in conventional electrolysis is the dependence on seamless energy input, while the intermittent power supply like sunlight or wind can significantly hamper the energy production [20, 21, 22]. The frequent variations in electricalpower can reduce the efficiency of electrolyzer, increase energy losses, and accelerate electrode degradation. Furthermore, OER is inherently sluggish, which can limit the efficiency of electrolyzer, cause catalyst degradation, and limit the durability of electrolyzers.

To overcome these limitations, recent developments have enabled the “decoupling” of the entire process through the use of redox mediators or supercapacitive electrodes for capacitive charge storage while temporally separating hydrogen and oxygen production on the bifunctional electrode [23, 24, 25, 26, 27]. The concept of decoupled water electrolysis (DWE) was first proposed by Symes and Cronin in 2013 [28], with notable advancements achieved since then [23, 24, 29, 30]. While DWE shows promise for further development, systematic reviews focusing on different categories of redox mediators and the possibility of future development remain limited. Furthermore, other upcoming decoupling strategies of water electrolysis that are being upscaled worldwide are also missing in the literature.

Here, we present an in‐depth discussion of representative redox mediators, membranes, and catalysts employed in this emerging approach. The identified research gaps are illustrated in Figure 1. Furthermore, we examine the operating mechanisms, advantages, and limitations of these redox‐decoupled systems. The concept of designing advanced decoupling agents is then emphasized, followed by a comprehensive assessment of the key challenges and prospects for redox‐mediated water splitting. The recent development of a membrane‐free supercapacitive electrolyzer represents a significant advancement towards the practical implementation of DWE system. By employing the capacitive half‐cells for temporal separation of the HER and OER, the DWE system enables continuous hydrogen production with high gas purity without the need for conventional ion‐exchange membranes, thus successfully reducing issues with gas crossover, membrane degradation, and system complexity. The commercial potential of this technology is demonstrated by the Swedish patent “*Electrochemical System Comprising Two Half‐Cells and Method for Electrochemical Production of Gas”* (Publication number: SE 546000), filed in 2021, which is being scaled to industrial green hydrogen production, highlighting the growing technological maturity and commercialization prospects of DWE [23, 24]. In 2023, Guo et al. employed supercapacitors as solid‐state redox mediators (SRMs), where Na_0.5_MnO_2_ was used as a pseudocapacitor and active carbon was used as a double layer capacitor for decoupling the OER and HER at current density of 1000 mA/cm^2^ with 8000 cycle life [31].

**FIGURE 1:**
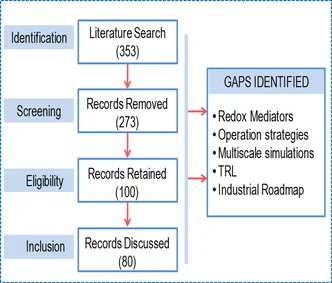
PRISMA flow chart. Flow diagram illustrating the search and selection process of articles on DWE following a systematic review approach.

## 2 Water Electrolysis Basics

### 2.1 Conventional Electrolysis

In conventional water electrolysis, a pair of electrodes is placed in a water‐based electrolyte, and a voltage adequate to decompose water into its fundamental constituents—hydrogen and oxygen, is applied to the electrodes. The complete process of water electrolysis involves the coupling of a reduction reaction at the cathode and an oxidation reaction at the anode. The schematic representation of conventional water electrolysis is given in Figure 2a. In the process, both electron flow and ion movement are necessary to keep the system electrically balanced and allow the reactions to continue. During electron movement through external circuits, water molecules are oxidized at anode, releasing electrons. Equation (1) portrays the oxidation half‐reaction of water in an acidic medium, commonly referred to as the OER. These released electrons cannot remain at the anode; they are driven through the external circuit towards the cathode, where they participate in the reduction reaction. As electrons leave the anode, positive charges (H^+^ ions) from acidic electrolyte accumulate near the anode. In order to maintain the electroneutrality, these H^+^ ions diffuse to the cathode and combine with the arriving electrons to form hydrogen gas. Equation (2) illustrates the reduction half‐reaction of water in an acidic medium, i.e., HER. Equation (3) represents the overall electrochemical dissociation of water in acidic environment, combining the Equations (1) and (2) [32].

(1) $$\text{Anode}:\;H_{2}O\;\to \;2H^{+}+2e^{-}+½O_{2}$$

(2) $$\text{Cathode}:\;2H^{+}+2e^{-}\;\to \;H_{2}$$

(3) $$\text{Overall}:\;H_{2}O\;\to \;H_{2}+½O_{2}$$

**FIGURE 2:**
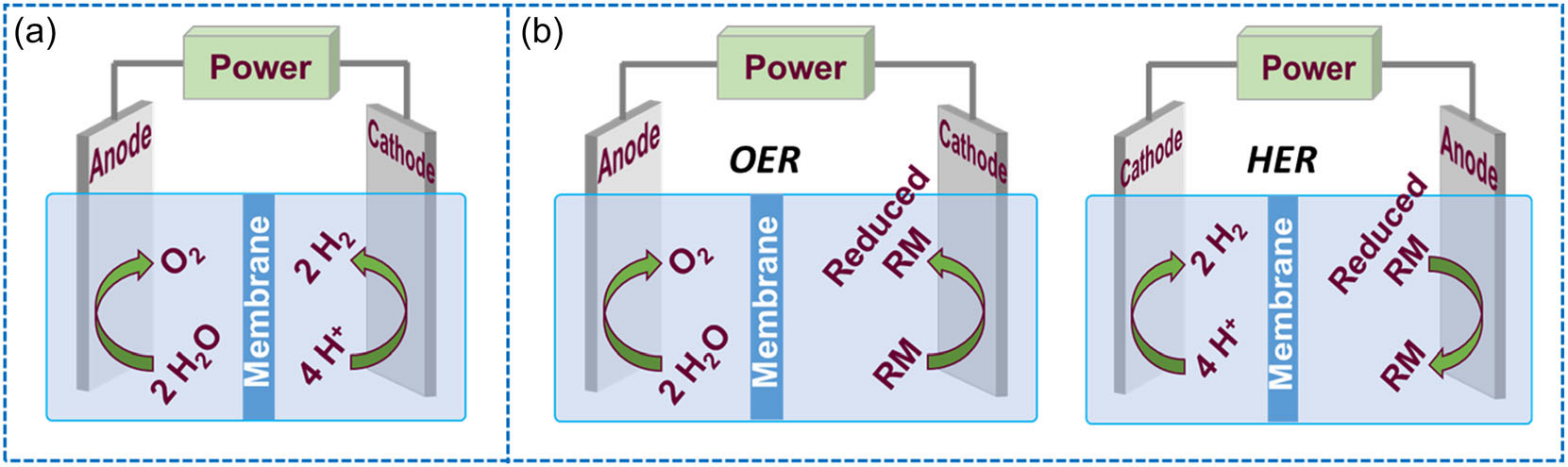
Schematic representation. (a) Conventional electrolysis. (b) DWE: OER and HER.

Similar electrochemical processes occur in alkaline media, where hydroxide ions (OH^−^), instead of protons (H^+^), act as the primary charge carriers. In alkaline water electrolysis, at the cathode, water molecules gain electrons and are reduced to hydrogen gas and hydroxide ions (Equation (4)), while at the anode, hydroxide ions are oxidized to release oxygen gas and electrons (Equation (5)). Equation (6) represents the overall water splitting process in alkaline media [32].

(4) $$\text{Cathode}:\;2H_{2}O+2e^{-}\;\to \;H_{2}+2{\mathrm{OH}}^{-}$$

(5) $$\text{Anode}:\;4{\mathrm{OH}}^{-}\;\to \;O_{2}+2H_{2}O+4e^{-}$$

(6) $$\text{Overall}:\;2H_{2}O\;\to \;2H_{2}+O_{2}$$

The theoretical minimum voltage required for splitting water is 1.23 V under standard conditions; however, practical electrolysis generally demands higher input due to overpotentials associated with both reaction kinetics and resistances within the electrolyzer setup [33].

However, the large‐scale application of conventional electrolysis is still limited by the poor operational stability and durability of electrolyzers. The commercial conventional electrolyzer technologies follow membrane/diaphragm‐bound strategy to separate the produced gases, but their high costs limit their commercial application. Motealleh et al. reported the exceptional stability of AEM electrolyzer for more than 11,000 h at 1000 mA/cm^2^ current density [34]. Nevertheless, many reported AEM electrolyzer still exhibit operational lifetimes below 3000 h due to membrane/diaphragm or electrode degradation [35]. Maoulida et al. systematically classified membrane degradation into three major categories: electrical, thermal, and dynamic operational degradation [36]. In conventional electrolyzers operated at high current density, the interfacial resistance between the catalyst layer and the porous membrane increases significantly with operation time, leading to higher operating voltages. Elevated operating temperature promotes membrane thinning, pinhole formation, and gas crossover through mechanical creeping that eventually reduces the device life‐time. Furthermore, intermittent ON/OFF operation associated to renewable energy sourcesinduces thermal and pressure fluctuations, catalyst dissolution, and subsequent accumulation on the membrane's surface, which result in voltage drifts. In direct seawater electrolysis, the corrosion effect is more significant as the chloride ions promote chlorine evolution, damaging the metallic components in the electrolyzer, as well as the catalysts and membranes, thus shortening the operational lifetime and reducing overall efficiency.

Although DWE offers significant advantages over conventional electrolysis, these benefits are highly dependent on the specific system architecture. Several DWE configurations have demonstrated reduced gas crossover, enhanced operational flexibility, and improved safety without a membrane requirement or any electrolyte management. Many DWE systems still require membranes, separators, auxiliary reactors, or careful handling of redox mediator and electrolyte management. In addition, the extent of improvements in durability, energy efficiency, and compatibility with intermittent renewable energy varies considerably with mediator chemistry, reactor design, and operating conditions. A membrane‐free system, solution‐phase redox mediator (SLRM), SRM, or dual‐cell DWE systems possess distinct advantages but also have some inherent limitations. Therefore, the advantages of DWE systems can be interpreted as architecture‐specific, and their effectiveness depends on underlying system design and operating conditions.

### 2.2 Decoupled Electrolysis Through Redox Coupling

In DWE through redox coupling, the OER is coupled with the reduction of redox mediator (M), rather than reduction of water molecules to produce H_2_, upon employment of a particular redox potential (Equations (7)–(12)). The decoupling process allows the two half‐reactions to be separated and facilitates the acceleration of the HER, as per Equations (9) and (12).

(7) $$\text{Cathode}:\;2H^{+}+2e^{-}\;\to \;H_{2}$$

(8) $$\text{Anode}:\;2M^{-}\;\to \;2M+2e^{-}$$

(9) $$\text{Overall}:\;2H^{+}+2M^{-}\;\to \;H_{2}+2M$$

(10) $$\text{Cathode}:\;2M+2e^{-}\;\to \;2M^{-}$$

(11) $$\text{Anode}:\;H_{2}O\;\to \;2H^{+}+2e^{-}+½O_{2}$$

(12) $$\text{Overall}:\;2M+H_{2}O\;\to \;2M^{-}+2H^{+}+½O_{2}$$

The overall reaction (combination of Equations (9) and (12)) corresponds to the conventional water‐splitting process, producing H_2_ and O_2_ as the final outputs. The mediator undergoes repeated cycling between different oxidation states, functioning similar to a redox‐flow battery (RFB) when in liquid form or to a solid‐state battery when in solid form. In both traditional and DWE, the only inputs are water and electrical energy, while the exclusive outputs are hydrogen and oxygen gases. As presented, Equations (9) and (12) are not separately balanced with respect to proton exchange, indicating that the pH will change if Equation (9) occurs without Equation (12) (and the other way around). Nevertheless, pH is predominantly governed by the composition of electrolyte solution, and the actual pH fluctuation during isolated operation of DWE systems relies on both the electrolyte's initial pH and total charge transferred during operation. Additionally, Equations (9) and (12) can be modified slightly to account for mediators involved in proton uptake and release during cycling (Equations (13)–(18)), as described in Equations (3) and (4).

(13) $$\text{Cathode}:\;2H^{+}+2e^{-}\;\to \;H_{2}$$

(14) $$\text{Anode}:\;2{\mathrm{MH}}_{2}\;\to \;2M^{*}+2H^{+}+2e^{-}$$

(15) $$\text{Overall}:\;2{\mathrm{MH}}_{2}\;\to \;H_{2}+2M^{*}$$

(16) $$\text{Cathode}:\;2M^{*}+2H^{+}+2e^{-}\;\to \;2{\mathrm{MH}}_{2}$$

(17) $$\text{Anode}:\;H_{2}O\;\to \;2H^{+}+2e^{-}+½O_{2}$$

(18) $$\text{Overall}:\;2M^{*}+H_{2}O\;\to \;2{\mathrm{MH}}_{2}+½O_{2}$$

The overall reactions (Equations (15) and (18)) remain identical to the conventional water‐splitting process, but the system preserves proton balancing. When mediators function in alkaline conditions, they undergo reactions analogous to Equations (15) and (18), but the charge‐balancing species are hydroxide ions (OH^−^) instead of protons (H^+^).

The OER and HER occur in separate spaces and intervals (either spatial separation or temporal separation, respectively) in DWE systems. The temporal separation in DWE technology has a few limitations, which cannot be underestimated. In a single DWE unit, H_2_ and O_2_ production follow one after another, which interrupts the continuous H_2_ production in the entire operating cycle, and sometimes H_2_ production time may be half of the total cycle time. But this limitation can be overcome by using multiple DWE units or operating different units alternately. But, these require additional electrodes, power systems, and electrolyte handling systems in a controlled manner, which may further increase the operational cost of the and system sizes. Although the membraneless DWE technology leads to reduction in cost, overall costs still depend on equipment add‐ons and system requirements needed for continuous gas production. The mediators used for decoupling the whole process should be stable both in the oxidized as well as in the reduced states. Additionally, the mediator should be a reversible redox pair, with electrochemical potential situated between the threshold potentials required to trigger the redox processes. Based on this concept, DWE can be recognized as the process when the final products of anodic and cathodic reactions should meet the following conditions: (i) the independent rates of anodic and cathodic reactions, (ii) the distinct time intervals of two reactions, and (iii) the reactions should be completed within the entirely separate electrochemical cells [37]. All the above‐discussed factors can place the DWE technology ahead of conventional electrolysis process. The schematic representation of DWE electrolyzer is given in Figure 2b [38, 39, 40, 41].

## 3 Examples of Decoupling HER/OER

### 3.1 Agents/Redox Mediators

#### 3.1.1 Soluble or Solution Dispersed Redox Mediators

##### 3.1.1.1 Polyoxometalates (POM) Redox Mediators

Redox mediators that dissolve or disperse readily in electrolytes provide significant advantages for uninterrupted operation and the development of hybrid devices, particularly in fluidic systems. In the first report, Symes and Cronin employed phosphomolybdic acid (PMA), POM (a 3D closed framework of transition‐metal oxyanions linked by oxygen), as a redox mediator operating in acidic conditions. Acting as electron‐coupled‐proton buffer (ECPB), PMA synchronously stores electrons and protons, adjusting the proton counts during water electrolysis. At the cathodic chamber, PMA undergoes protonation (Equation (19)) that reoxidizes to drive hydrogen generation (Equation (20)). This can enable decoupling of OER and HER, with reduced PMA remaining stable for days, sustaining HER efficiently. During the process of electrons and protons uptake, the POM enables the electrolyte to act as a buffer, and thus, the ECPB concept was introduced to other mediators with comparable redox properties [42].

(19) $$\text{ECPB}+H_{2}O\;\to \;[{\text{ECPB}}_{\left(2e-\text{reduced}\right)}]\;{\left[H^{+}\right]}_{2}+½O_{2}$$

(20) $$[{\text{ECPB}}_{\left(2e-\text{reduced}\right)}]\;{\left[H^{+}\right]}_{2}\;\to \text{ECPB}+H_{2}$$

Lu and coworkers extended the use of PMA in a DWE configuration employing a bipolar membrane, where PMA circulated as a mobile mediator, stabilizing pH during HER and OER and efficiently maintaining the proton balance [43]. In its 2e^−^ redox cycle, PMA enable temporal separation of O_2_ and H_2_ evolution in a near‐stoichiometric 1:2 ratio, showing high reproducibility over repeated cycles. The ECPB‐driven approach also facilitated 6e^−^ redox electrolysis for decoupled OER/HER using low‐cost earth‐abundant and noble‐metal‐free catalysts such as NiFe LDH/Ni foam (Nickel–Iron Layered Double Hydroxide grown on Nickel foam substrate) and Ni_3_S_2_/Ni foam (Nickel(II) sulfide grown on Nickel foam substrate), which exhibited lower voltage requirements compared to the classical HER electrodes of platinum (Pt) and OER electrodes of iridium oxide (IrO_2_) (Figure 3a) [43]. These noble‐metal‐free catalysts are well known for their high efficiency in water electrolysis [45, 46, 47, 48, 49]. Furthermore, PMA presents additional advantages, including multiple accessible redox peaks [50], commercial availability, and high solubility exceeding 0.5 M, making it a promising redox mediator for such systems. Transitioning between oxidized (H_3_PMo_12_O_40_) and reduced (H_5_PMo_12_O_40_) PMA, it stabilizes pH and resists reoxidation, enabling reliable O_2_/H_2_ separation, thus serving as a benchmark for the evaluation of emerging decoupling agents [42].

**FIGURE 3:**
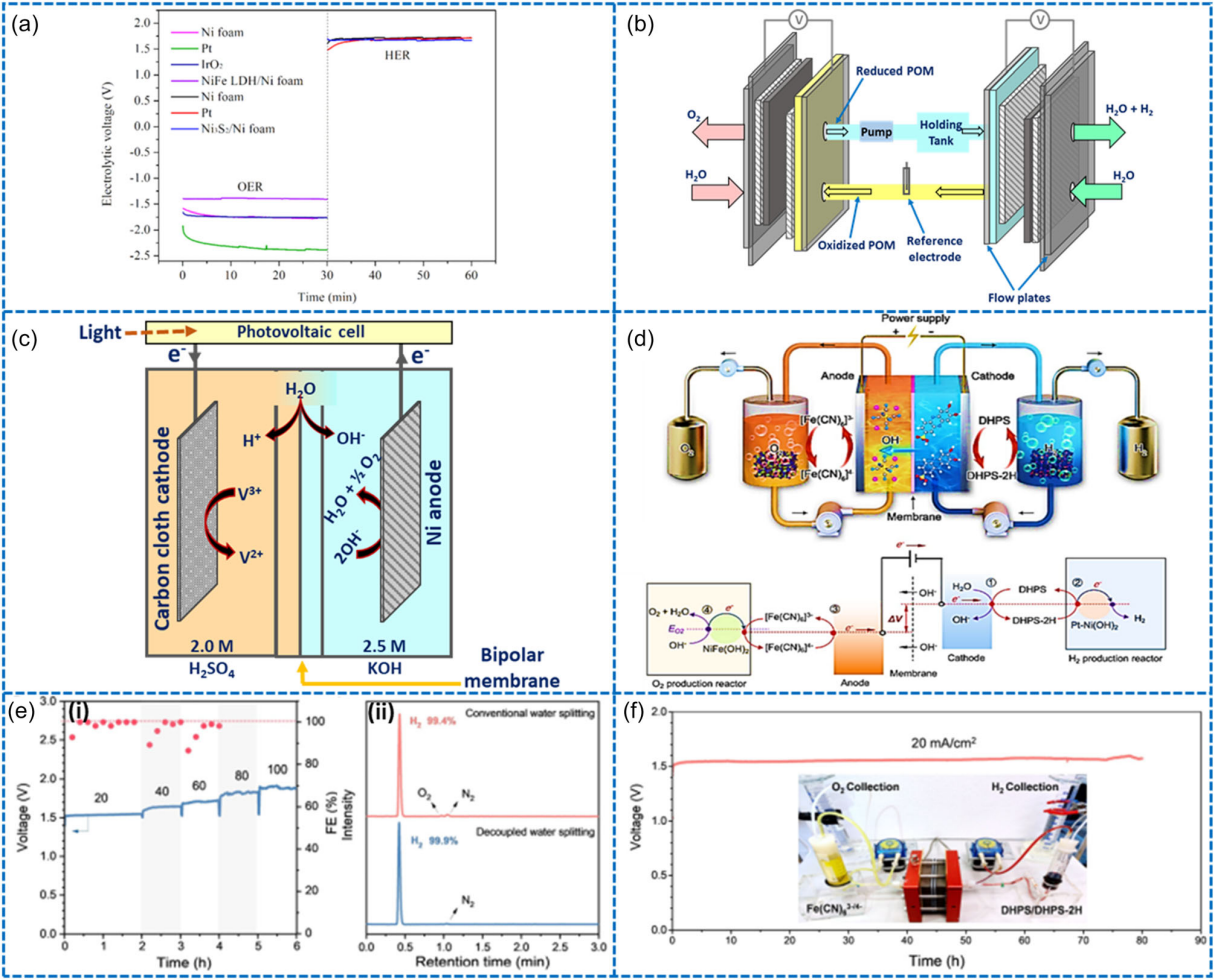
Experimental setups and characterizations of DWE systems. (a) A two‐electrode configuration of electron‐coupled proton buffer (ECPB)‐based water electrolysis with separated OER and HER at 50 mA/cm^2^ with 10 mmol of phosphomolybdic acid in one cell and 60 mL of 0.5 M NaOH aqueous solution in another. Reproduced with permission [43]. Copyright 2020, ACS. (b) A flow cell for [P_2_W_18_O_62_]^6−^ redox reactions. (c) A solar‐driven DWE using the V(III)/V(II) redox couple. (d) Schematic of a redox‐flow electrolytic configuration enabling spatial separation of O_2_ and H_2_. Reproduced with permission [44]. Copyright 2021, ACS. (e) System characterization: (i) voltage response and Faradaic efficiency at various current densities, (ii) GC spectra of gases in decoupled system versus conventional cell. Reproduced with permission [44]. Copyright 2021, ACS. (f) Voltage profile during continuous H_2_ production for 82 h at 20 mA/cm^2^ (the cell setup is shown in inset). Reproduced with permission [44]. Copyright 2021, ACS.

Decoupling HER and OER introduces an inherent energy penalty since the redox mediator undergoes both reduction and reoxidation, slightly lowering overall efficiency. Symes and Cronin reported ~87% efficiency at 100 mA/cm^2^, comparable to conventional electrolysis [28]. Using a 50:50 oxidized/reduced ECPB mixture to mimic cycling, the two‐step pathway required 2.94 V, compared to 2.55 V necessary for direct splitting, with the ~400 mV penalty mainly due to the ECPB overpotentials [28]. Rausch et al. proposed silicotungstic acid (STA) as an alternative mediator to mitigate this problem [51]. In acidic media, STA displays cathodic redox peaks (*E*_½_ = +0.01, −0.22 V vs. SHE), enabling two‐electron reduction at the electrodes without parasitic HER. The unstable reduced STA spontaneously reoxidized at Pt/C electrodes, releasing H_2_ without added electrochemical input, thereby enhancing DWE efficiency that was validated in a two‐chamber cell, where OER occurred at a platinum mesh anode and STA reduction at a carbon cathode [42, 51]. STA‐mediated system is reported to achieve 100% Faradaic efficiency (FE) for OER with negligible H_2_ traces. Reduced‐STA, when combined with the HER catalysts, enabled spontaneous hydrogen release. Carbon‐supported platinum markedly outperformed molybdenum (Mo) and nickel (Ni) catalysts, delivering hydrogen generation rates rivaling advanced PEM electrolyzers. This improvement arises because Pt/C in the decoupled setup operates without direct electrical contact, allowing a 3D catalytic configuration, unlike the conventional 2D arrangement constrained by current collectors [51]. The STA‐mediated electrolysis was adapted into a PEM flow‐cell, achieving stable operation over 25–500 mA/cm^2^ [52]. Hydrogen contamination in the O_2_ output remained <0.4% even at 25 mA/cm^2^, whereas a conventional PEM electrolyzer produced >2% H_2_ under similar conditions, close to the 4 vol% safety threshold [53]. Durability of the electrolytic cell was assessed for 200 h by monitoring fluoride ions, as a marker of sulfonated tetrafluoroethylene‐based fluoropolymer‐copolymer (Nafion) degradation [52]. The STA‐mediated system showed reduced membrane decomposition versus coupled systems, attributed to spatial HER/OER separation lowering reactive oxygen species and slowing ion‐exchange membrane deterioration [54]. Symes and group used STA mediator in DWE and achieved high current density of up to 1350 mA/cm^2^, successfully competing with commercial AEM or PEM electrolyzers. The STA‐mediated decoupled system showed 99.9% decoupling efficiency, 43% energy efficiency, and 60 times higher turnover at high current density [55].

In addition to PMA and STA, other ECPB mediators such as phosphotungstic acid (PTA) and silicomolybdic acid (SMA) have been examined for spontaneous HER. MacDonald and coworkers investigated these mediators with Mo‐ and Ni‐based electrocatalysts molybdenum carbide (Mo_2_C), molybdenum disulfide (MoS_2_), or nickel phosphides (Ni_2_P, Ni_5_P_4_) in decoupled HER. One‐electron and two‐electron reductions of 1 M mediator solutions were reported, followed by exposure to catalysts for HER evaluation. Amongst the catalysts studied, Ni_5_P_4_ and Mo_2_C yielded the highest HER activity, though still lower than Pt/C benchmarks [56].

Chen et al. subsequently transformed the ECPB to enhance energy density [57]. Preceding inorganic ECPB systems were capable of accepting two electrons and two protons, but several reports demonstrate the uptake of more than two electrons and protons by the redox mediators [58, 59]. Lei and coworkers performed a relative study of other POMs containing tungsten such as [XW_12_O_40_], with the “X” being Zn^2+^, P^5+^, or Si^4+^. To assess the impact of the main heteroatom on POM clusters’ redox properties, bulk electrolysis was employed, validating that both PTA and STA experience two‐electron reduction processes [60]. However, the Zn^2+^ counterpart, tungstozincic acid (H_6_ZnW_12_O_40_), was found to be capable of four electrons uptake under similar electrochemical conditions. No pH changes were noticed during either the acceptance of electrons or subsequent electron donation, indicating that H_6_ZnW_12_O_40_ functions effectively as an ECPB. The tungstozincic acid cluster as decoupling agent demonstrated impressive performance in electrochemical water splitting and effectively decoupled the two redox processes: OER and HER. The redox mediator was reported to exhibit enhanced stability and was run more than 200 times in an uninterrupted flow setup, with a Coulombic efficiency per run exceeding 90%. The easily reducible polyoxoanion‐based ECPB, for example, the lithium *α*‐Dawson polyoxotungstate (Li_6_ [P_2_W_18_O_62_]), could accept up to eighteen electrons and eighteen protonsin a reversible manner, as reported by Chen et al. [57]. To establish the decoupling nature of Li_6_ [P_2_W_18_O_62_] cluster, the researchers set up a dual electrochemical flow cell setup (Figure 3b) [57]. In anodic compartment with IrO_2_ anode, oxidation of water occurred at a fixed current density, while reduction of Wells–Dawson phosphotungstate anion [P_2_W_18_O_62_]^6–^ occurred at cathode. The reduced POM‐redox mediator was redirected to the anodic compartment for further reduction of the POM that, upon reduction to a desired extent, was then transferred to the cathodic compartment, where it was reoxidized, accompanied by HER. The electrons transferred during the reduction of POM were compared to the electrons gained during reoxidation, revealing the reversibility pertaining to the energy storage mechanism. The authors further reported that at 0.1 M concentration of Li_6_ [P_2_W_18_O_62_], the recovered charge was 96%. This corresponded to approximately 17.2 electrons regained for each 18‐electron reduction of every polyoxoanion. The “lost” charge in this occasion could be attributed to the two factors: (i) contact of 18 electron reduced POM with trace amount of oxygen in the setup, which resulted in spontaneous reoxidation of the POM, and (ii) presence of metallic impurities resulted in trace amount of hydrogen generation from the reduced POM. Although the polyoxoanion was reducible by up to 32 electrons, the Coulombic efficiency dropped significantly beyond the 18‐electron reduction, revealing that polyoxoanions reduced beyond this level were unstable in terms of spontaneous hydrogen production. The Coulombic efficiency was reported to be 95% after 100 cycles, indicating the potential of this POM as a competent redox mediator for constant flow DWE. This decoupling agent successfully produced hydrogen at a rate of 1.31 L/min/h; when carbon‐supported platinum electrode was employed, the H_2_ production rate was typically much higher compared to conventional PEM electrolyzer (0.02–0.04 L/min) [61].

##### 3.1.1.2 Transition Metal‐Based Redox Mediators

DWE systems modeled after RFB were engineered using standard flow battery electrolytes. Amstutz and coworkers in 2014 presented a vanadium‐derived catholyte and a cerium‐derived anolyte for DWE [62]. In this configuration, electrochemical reduction was processed for V(III) in sulfuric acid at graphite felt electrode, whose potential (−0.26 V vs. SHE) is sufficiently negative to drive spontaneous hydrogen evolution. Upon transferring the reduced V(II) solution to an auxiliary catalytic reactor, hydrogen production was accomplished with a FE of up to 96%, employing a Mo_2_C electrocatalyst. At the anode, Ce(III) was oxidized to Ce(IV), the potential of which in acidic medium is more positive than what is required for water oxidation, enabling OER in the presence of ruthenium dioxide (RuO_2_) nanoparticles with 78% ± 8% FE. Reduced performance was linked to Ce(IV)‐induced RuO_2_ degradation. To overcome this, vanadium components (V^5+^/V^4+^) were incorporated, enabling both rechargeable cycling and energy‐to‐hydrogen conversion, similar to [P_2_W_18_O_62_]^n−^ systems [57].

Ho et al. investigated the V(III)/V(II) electron‐transfer couple in a photo‐driven DWE system [63]. Since most earth‐abundant OER catalysts are stable in alkaline media, whereas V(III)/V(II) is soluble only in acidic conditions, a bipolar membrane was used to sustain a strong pH gradient between the electrodes. This enabled OER in 2.5 M KOH at a nickel mesh anode and HER in 2.0 M H_2_SO_4_ at a carbon cloth cathode (Figure 3c) [63]. Reduced V(II) was subsequently transferred to a Mo_2_C reactor for hydrogen generation, achieving Faradaic efficiencies up to 87%, depending on the extent of V(III) reduction. Zhang et al. introduced a new strategy of employing two types of redox mediators in the DWE process by employing two redox mediators: 7,8‐dihydroxy‐2‐phenazinesulfonic acid (DHPS) in the cathode and Fe(CN)_6_^4−^ in anode of the dual‐compartment electrochemical cell. During electrolysis, water molecules were reduced at the cathode leaving behind OH^−^, which streamed into the anode via membrane while oxidizing the Fe(CN)_6_^4−^ to Fe(CN)_6_^3−^ in presence of nickel–iron‐based electrocatalyst. This led to the generation of molecular oxygen in the anode compartment, while at the cathode, DHPS underwent two electron reduction, enabling the hydrogen production in presence of platinum–nickel‐based electrocatalyst (Figure 3d). The decoupled system operated with DHPS reduction at the cathode and its catalytic dehydrogenation on Pt–Ni(OH)_2_ catalyst bed, releasing H_2_, while [Fe(CN)_6_]^3−^/^4−^ mediated O_2_ generation on NiFe(OH)_2_ at the anode (Figure 3e(i,ii)). A voltage plateau of 1.53 V with nearly 100% FE was achieved at 20 mA/cm^2^ and maintained for over 80 h without degradation. The average cell voltage gradually increased with current density, reaching 1.91 V at 100 mA/cm^2^, with Faradaic efficiencies recovering after initial attenuation. Gas analysis confirmed nearly pure H_2,_ thus suggesting negligible O_2_ crossover (Figure 3f) [44].

Goodwin and Walsh illustrated the decoupling of OER and HER employing the ferricyanide/ferrocyanide (Fe(CN)_6_^3−^/Fe(CN)_6_^4−^) redox pair in 0.1 M KOH within closed bipolar electrode (CBE) setup, incorporating Nafion membrane and Pt electrodes [64]. The bipolar electrode, fabricated from two carbon cloths connected by a wire, linked two separate dual‐compartment cells (Figure 4a,b). In this setup, Fe(CN)_6_^3−^ was reduced while water was oxidized at one Pt electrode, whereas Fe(CN)_6_^4−^ oxidation at the opposite end drove H_2_ evolution. This configuration effectively separates OER/HER while suppressing gas crossover and enables operation with differing pH in each compartment. They further showed that the CBE could run in reverse as a fuel cell. As illustrated in Figure 4c(i–iii), LSV and polarization measurements revealed HOR/ORR behavior comparable to that of single cells, with a maximum power density of ~100 μW/cm^2^. A mixed‐pH fuel cell (acidic cathode and alkaline anode) produced 1.82 V and ~4.5 mW/cm^2^ (Figure 4c(iv)) [64].

**FIGURE 4:**
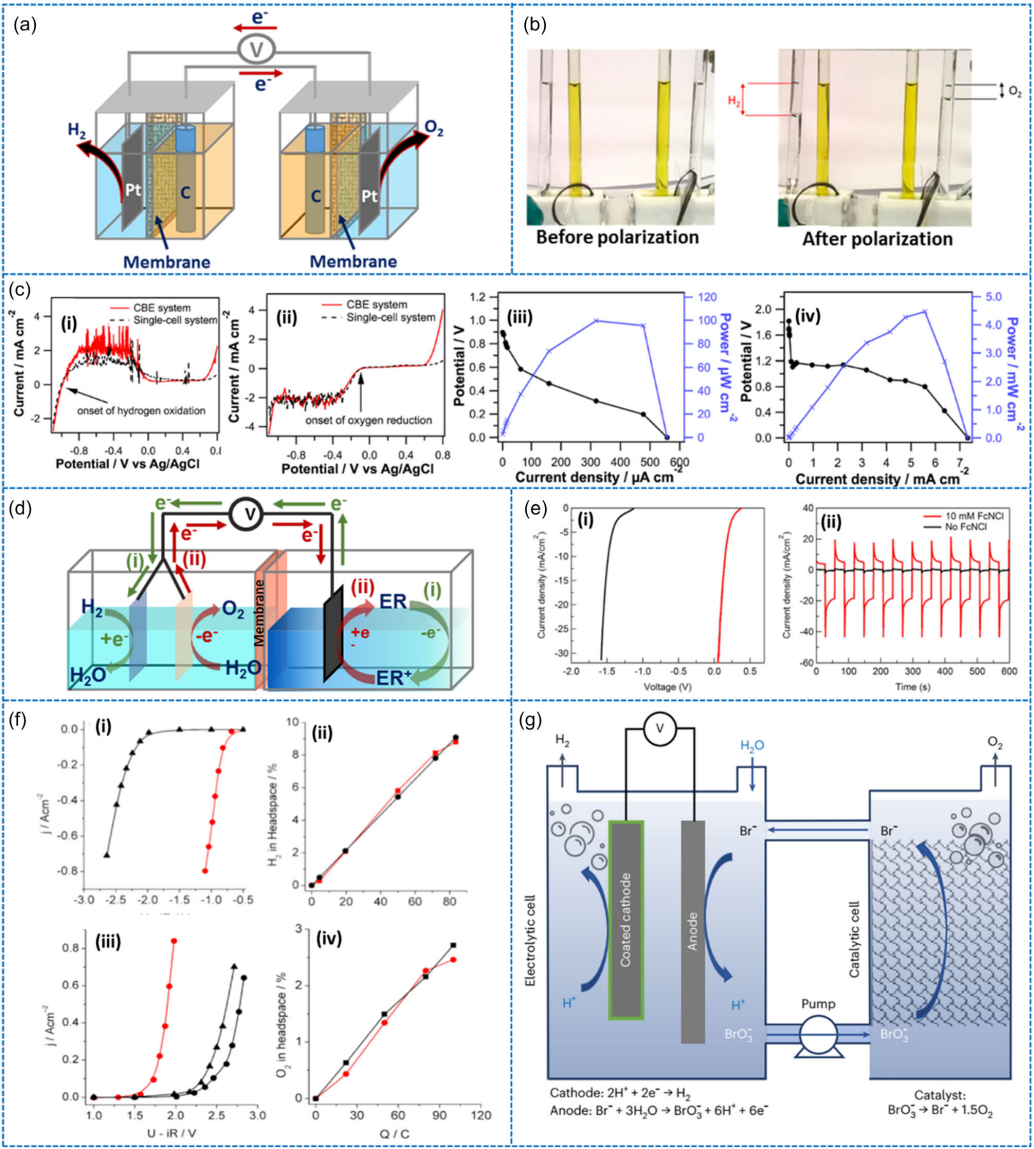
Electrolyzer designs and performance analyses. (a) Schematic closed bipolar electrode (CBE) setup. (b) CBE system showing before and after applying 3.7 V (colorless solution: 0.1 M KOH, yellow solution: 0.1 M KOH + 0.25 M K_3_Fe(CN)_6_ + 0.25 M K_4_Fe(CN)_6_). Reproduced with permission [64]. Copyright 2017, ACS. (c) LSV: (i) H_2_‐loaded, (ii) O_2_‐loaded 0.1 M KOH, (iii) polarization curve in CBE with O_2_/H_2_ electrodes (black) and power–potential (blue), and (iv) CBE with H_2_SO_4_/KOH (black) and power‐potential plot (blue). Reproduced with permission [64]. Copyright 2017, ACS. (d) Schematic representation DWE system with sequential HER and OER. (e) Electrocatalytic HER driven by a photovoltaic cell under sunlight: (i) LSV under sunlight‐with (red) and without (black) photovoltaic assistance, and (ii) time‐dependent current density under chopped sunlight. Reproduced with permission [65]. Copyright 2018, Elsevier. (f) (i) i–v curves showing H_2_ evolution in two‐electrode‐two‐compartment cell: the two compartments are loaded with ECPB/ECPB*+H_3_PO_4_ and pure H_3_PO_4_, respectively (red) or pure H_3_PO_4_ and H_3_PO_4_, respectively (black), (ii) calculated (black) versus measured (red) H_2_ content determined by GCHA, (iii,iv) O_2_ evolution: likewise (f), (i,ii) black lines with triangles and circles are, respectively, the data collected in Pt mesh and carbon cloth counter electrode. Reproduced with permission [66]. Copyright 2013, ACS. (g) Schematic representation of Br^−^/BrO_3_^−^ mediated DWE system. Reproduced with permission [67]. Copyright 2024, Springer Nature.

Li et al. examined the application of a ferrocene derivative, (ferrocenylmethyl)trimethylammonium chloride (FcNCl or C_14_H_20_ClFeN), as a redox mediator for decoupling HER and OER [65]. A two‐compartment H‐cell was employed for water electrolysis where one chamber was charged with counter electrode (carbon in 50 mM FcNCl + 0.5 M sodium sulphate (Na_2_SO_4_)), and the other compartment employed Ni_2_P/NF for HER and bare NF for OER. Electrical connection through the counter electrode enabled decoupling (Figure 4d). 100% FE was reported with stability over 20 cycles, despite pH oscillations (rising to 9 during HER and returning to 6.5 during OER), and no loss in redox activity was noticed. Furthermore, solar‐driven HER was demonstrated by coupling with a ~1.6 V photovoltaic cell, enabling bias‐free H_2_ production in near‐neutral electrolyte. As shown in Figure 4e(i,ii), photocatalytic HER was verified through LSV and chopped‐light experiments, with visible H_2_ evolution.

##### 3.1.1.3 Organic Redox Mediators

During the initial advancement of DWE, investigators observed that separation of HER and OER via redox mediators mirrored mitochondrial electron transport that depends on quinone‐based electron‐proton shuttles. Building on this concept, Rausch et al. introduced potassium hydroquinone sulfonate (PHQS), where a sulfonate group enhanced aqueous solubility in both oxidation states. As shown in Figure 4f(i), current density of 500 mA/cm^2^ was achieved at 0.98 V with ECPB, whereas >2.5 V was required without it, necessitating Pt mesh instead of carbon felt. Gas chromatography confirmed H_2_ evolution with a FE of 98% ± 7% (Figure 4f(ii)), while O_2_ formation from water splitting was negligible. Hydroquinone sulfonate effectively minimized pH shifts, validating its role as an ECPB by shuttling protons and electrons. Figure 4f(iii,iv) further shows O_2_ evolution at 500 mA/cm^2^ and 1.90 V, with 91% ± 5% efficiency, confirming efficient HER/OER decoupling [66].

Kirkaldy et al. investigated anthraquinone‐2,7‐disulfonic acid (AQDS) as a redox mediator for DWE [58]. A reversible redox potential of +0.21 V versus SHE, with only 5.75% capacity loss after 100 cycles in 1 M H_2_SO_4_, was reported from cyclic voltammetry, and close to 100% FE was achieved for both HER and OER. AQDS‐mediated dual‐flow cell demonstrated practical feasibility in water electrolysis, producing 1000 mA/cm^2^ current density with energy efficiency of 62.63%.

In addition to three main categories of soluble or solution‐dispersed redox mediators, another example of soluble redox couple (SRC) “Br^−^/BrO_3_^−^” was reported for DWE process [67]. This SRC is capable of storing and releasing oxygen in turns. In Br^−^/BrO_3_^−^ mediated process, hydrogen is generated at the cathode, while Br^−^ gets re‐oxidized at the anode (Equations (21) and (22)). The schematic representation of the reactor is given in Figure 4g.

(21) $$2H^{+}+2e^{-}\;\to \;H_{2}$$

(22) $$2{\mathrm{Br}}^{-}\;\to \;{\mathrm{Br}}_{2}+2e^{-}$$

The bromine molecules produced at the anode react with water molecules to produce hypobromous acid (HBrO), and eventually hypobromite anions (BrO^−^) and bromate anions (BrO_3_^−^) are formed, as per Equations (23)–(25).

(23) $${\mathrm{Br}}_{2}+H_{2}O⇌\text{HBrO}+H^{+}+{\mathrm{Br}}^{-}$$

(24) $$\text{HBrO}⇌H^{+}+{\mathrm{BrO}}^{-}$$

(25)

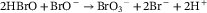




The overall anodic reaction is Equation (26) (summarizing Equations (22)–(25))



(26)

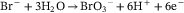




However, at the cathode, the reduction of hydrogen intermediate leads to the generation of hydrogen gas (Equation (27)).

(27) $$2H^{+}+2e^{‐}\;\to \;H_{2}\;(g)$$

Despite successful hydrogen production, some unwanted side reactions (Equations (28)–(32)) also occur at the anode and hamper the OER process because of the presence of HBrO and BrO^–^ in the reaction chamber.

(28) $$6{\mathrm{BrO}}^{-}+3H_{2}O\;\to \;2{\mathrm{BrO}}_{3}^{‐}+6H^{+}+4{\mathrm{Br}}^{-}+1.5O_{2}+6e^{-}$$

(29) $$\text{HBrO}+2e^{-}\;\to \;{\mathrm{Br}}^{-}+{\mathrm{OH}}^{-}$$

(30) $${\mathrm{BrO}}^{-}+H_{2}O+2e^{-}\;\to \;{\mathrm{Br}}^{-}+2{\mathrm{OH}}^{-}$$

(31)

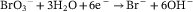






(32)

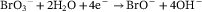




OER is severely suppressed by the operational conditions of 60 °C and pH 8 (Equation (28)), which yields 100% FE for bromate production (Equation (26)). To generate hydrogen effortlessly, sodium dichromate is added to the sodium bromide aqueous electrolyte solution, which suppresses the cathodic backward reactions (Equations (29)–(32)) while reducing the dichromate anions and subsequent deposition of chromium hydroxide layer on the cathode. The chromium hydroxide layer is semipermeable and protects the cathode from side reactions. This process leads to significant improvement in FE for HER from 10% ± 1% (without sodium dichromate) to 98% ± 2% (with 3.8 mM sodium dichromate) without using any membrane in a typical decoupled DWE system. Notably, the addition of sodium dichromate successfully suppresses the undesired side reactions, therefore leading to a hindrance‐free OER at the anode as per Equation (33).

(33) $$\text{2}{\text{BrO}}_{3}^{‐}\;\text{→}\;\text{2}{\text{Br}}^{-}+\text{3}O_{\text{2}}$$

#### 3.1.2 Solid Redox Mediators

##### 3.1.2.1 Transition Metal‐Based Redox Mediators

Certain features were recommended for efficient ECPB's, though these were guidelines rather than strict rules which interestingly were overlooked by researchers. For instance, while water solubility was once considered essential, the development of SRMs proved it unnecessary. Unlike soluble mediators, solid‐state systems avoid membranes or separators and instead utilize an auxiliary electrode. Still, mediators must feature at least one reversible redox pair between HER and OER potentials, exhibiting stability in both states, and preferably be inexpensive, stable, and easily synthesized. The solid‐state DWE was first reported in 2016 by Chen and coworkers employing nickel‐based Ni(OH)_2_/NiOOH auxiliary electrode [68]. The gas production using Ni(OH)_2_/NiOOH auxiliary electrode in DWE system proceeded with the following Equations (34) and (35).

(34) $$\mathrm{Ni}{\left(\mathrm{OH}\right)}_{2}\;\to \;\text{NiOOH}+½H_{2}$$

(35) $$2\text{NiOOH}+H_{2}O\;\to \;2\mathrm{Ni}{\left(\mathrm{OH}\right)}_{2}+½O_{2}$$

In this process, the cell performance was examined using chronopotentiometry measurement in between 100 and 500 mA applied current. The chronoamperometry performance at 200 mA current showed better performance, as shown in Figure 5a. The redox process employing Ni(OH)_2_/NiOOH auxiliary electrode comprises two steps: step 1 (H_2_ production), where a cell voltage obtained is ~1.6 V, resulting from the anodic potential of 0.5 V (vs. Hg/HgO) for Ni(OH)_2_ oxidation and the cathodic potential of −1.1 V (vs. Hg/HgO) for H_2_O reduction, and in step 2 (O_2_ production), the cell voltage obtained is 0.4 V, resulting from anodic oxidation of OH^−^ (0.7 V vs. Hg/HgO) and the cathodic reduction of NiOOH (0.3 V vs. Hg/HgO). At the end of step 2, the voltage increases, which is the indication of complete reduction of NiOOH to Ni(OH)_2_. The process is required to end automatically within 600 s, with Coulombic efficiency ≈100%. With this technology, the DWE cell demonstrates consistent and reproducible H_2_ and O_2_ generation of more than 20 repetitive cycles, where the distinct HER (step 1) and OER (step 2) necessitate different driving voltages, indicating that renewable energy sources like solar or wind can be strategically employed according to their variable output profiles (Figure 5b).

**FIGURE 5:**
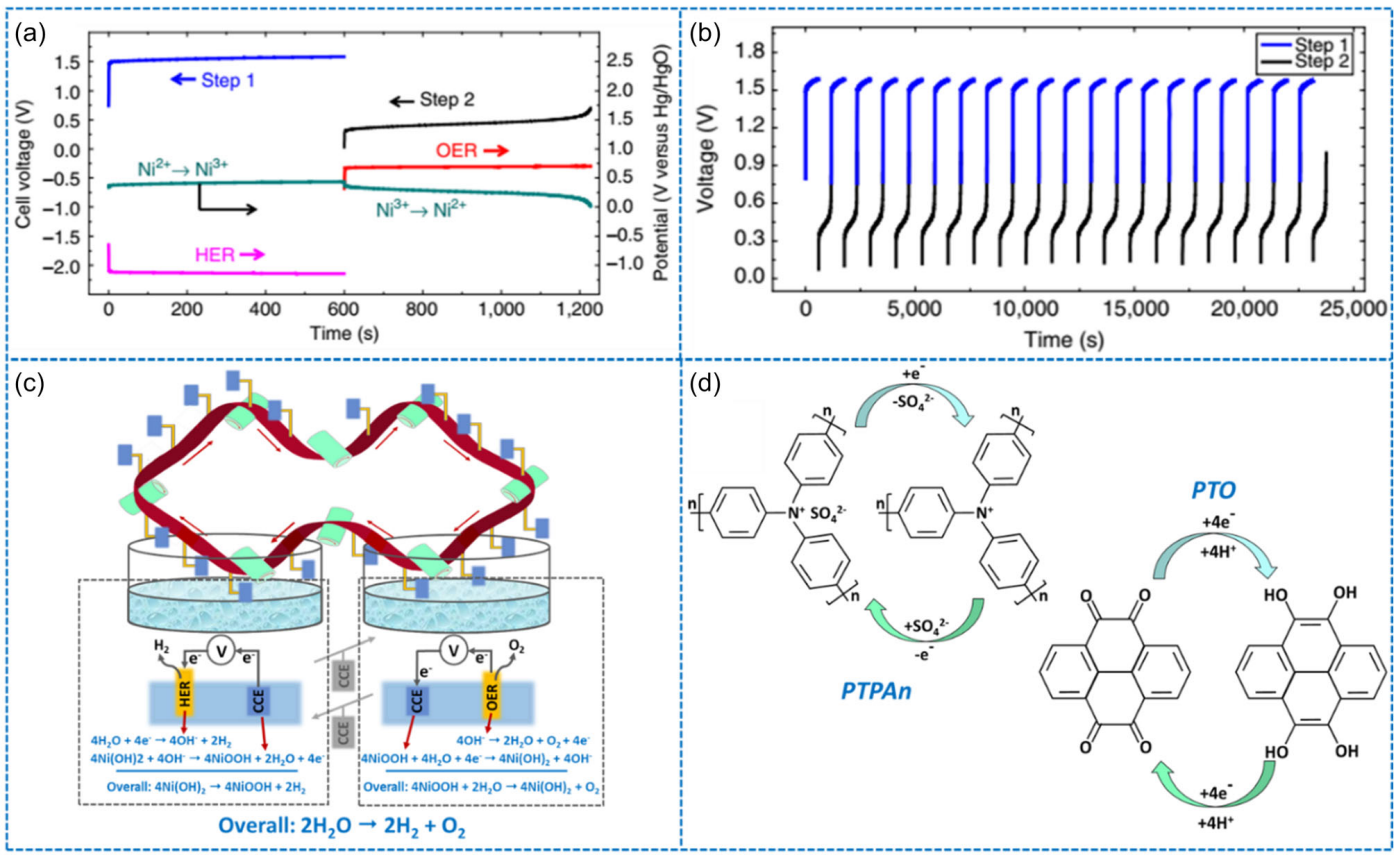
DWE performance study in alkaline medium, electrolyzer's design, decoupling agents. (a) Chronopotentiometry (cell voltage vs. time) at 200 mA shows for HER (blue), OER (black), and other electrodes. Individual electrode potentials (potential vs. time) are also shown: HER (pink), Ni(OH)_2_ (green), and OER (red). Reproduced with permission [68]. Copyright 2016, Springer Nature. (b) Chronopotentiometry cycle for alternating H_2_O_2_ generation at 200 mA with 600 s step intervals. Reproduced with permission [68]. Copyright 2016, Springer Nature. (c) Schematic representation of Ni(OH)_2_/NiOOH auxiliary electrode‐driven DWE employing a cable car‐style mechanism. (d) The PTPAn redox couple employed by Ma et al. [69] and the PTO redox couple employed by Xia's group [70].

Long et al. described a comparable apparatus designed to separate HER and OER processes, utilizing Ni(OH)_2_/NiOOH‐based auxiliary electrode system functioning like a cable car that continuously transports multiple auxiliary electrodes between the compartments (Figure 5c) [71]. The operation required an external voltage of at least 1.4 V for HER, while OER proceeded with less than 0.9 V at a current density of 50 mA/cm^2^. At 20 mA/cm^2^, OER demanded was under 0.5 V. The researchers reported a FE of 98% with 100% hydrogen purity. The total applied voltage of ≈2.0 V aligns with the pioneering study conducted by Chen et al. [68]. Rothschild et al. employed the Ni(OH)_2_/NiOOH mediator in a CBE system to decouple OER and HER under alkaline conditions [25]. In dual‐cell electrolyzer setup using 1 M NaOH and a copper wire, effectively produced green hydrogen for 20 h with nearly 100% FE. The system was highly efficient and performed electrolysis for 125 h, though the performance was slightly reduced by partial charging process. Solar‐driven operation integrated with silicon photovoltaic module achieved solar‐to‐hydrogen efficiency of 7.5%, which could be compared with next‐generation solar water‐splitting devices [72, 73, 74, 75, 76].

The spontaneous OER and electron losing phenomenon were demonstrated by Dotan et al. and Symes et al. integrating Ni(OH)_2_/NiOOH redox mediator [77, 78]. Employing cobalt‐doped nickel hydroxide electrode (Ni_0.9_Co_0.1_(OH)_2_) in 5 M KOH at 95 °C was highly effective to produce gases with 98.7% FE, highlighting the improved energy performance by exploiting waste heat for spontaneous OER.

MnO_2_ mediator electrode integrated three‐electrode system comprising Ni(OH)_2_ and La–Ni hydride electrodes was introduced by Choi et al. [79]. OER coupled with manganese dioxide or manganese(IV) oxide (MnO_2_) reduction occurred first, followed by manganese oxyhydroxide or manganite (MnOOH) oxidation with subsequent water reduction to generate hydrogen in separate steps (Equations (36) and (37)).

(36) $$2{\mathrm{MnO}}_{2}+H_{2}O\to 2\text{MnOOH}+½O_{2}$$

(37) $$2\text{MnOOH →}\;2{\mathrm{MnO}}_{2}+H_{2}$$

At 25–60 °C, a 10 h constant current test at 5 mA confirmed successful decoupling of HER and OER, though efficiency was reduced due to MnO_2_ overpotentials. Liu et al. later demonstrated sunlight‐driven decoupling using a Ni–Mn–P/Ni_2_P pseudocapacitor system, achieving HER–OER cycling with minimal additional voltage input [80].

*α*‐MoO_3_ is recognized as an efficient redox mediator in DWE. This layered metal oxide possesses ultrafast proton‐transfer property owing to hydrogen bond network in the lattice oxygen arrays of H_*x*_MoO_3_, while facilitating fast hydrogen production in acidic electrolyte (10 M H_3_PO_4_). The efficient redox mediator achieved capacity of 204 mAh/g with 100% pure hydrogen, outperforming many benchmarked solid redox mediators. Despite the high efficiency, *α*‐MoO_3_ sometimes encounter parasitic side reactions, which significantly triggers the low performance in DWE. The parasitic side reactions initiate from over charging beyond optimal protonation (typically >0.45 V vs. Ag/AgCl in acidic electrolytes like 10 M H_3_PO_4_), facilitating the OER to occur on *α*‐MoO_3_ surface and lead to 10 or 20‐fold decrease in FE and mediator degradation. The process noticeably reduces the efficiency, scalability, and stability of the decoupled system. This challenge can be addressed by optimizing the potential window and electrolyte composition to achieve continuous hydrogen production [81].

##### 3.1.2.2 Solid Organic Redox Mediators

The separation of HER and OER was verified through polytriphenylamine (PTPAn) by Ma et al. [82]. PTPAn features a skeleton assembled from highly conducting poly(paraphenylene) backbones with electrically active polyaniline (PANI) moieties, endowing the decoupling agents with exceptional electron‐transfer properties and rendering it a highly effective redox mediator (Figure 5d) [79, 83, 84]. Cyclic voltammetry measurements revealed that PTPAn could exhibit two distinct redox couples, placing it among the onset potentials of HER and OER. The Faradaic efficiencies nearing 100% for OER and HER were achieved through galvanostatic electrolysis conducted at a steady current of 100 mA. Direct conversion of solar energy and water into hydrogen and oxygen with a conversion efficiency of 5.4% was demonstrated. Xia and coworkers described DWE employing pyrene‐4,5,9,10‐tetraone (PTO) where the two redox processes (HER and OER) were independently linked to the enolized PTO (Figure 5d) [69]. Unlike PTPAn, the PTO‐based redox mediator possessed reversible oxidation and reduction during its redox process, enabling it to potentially buffer the solution's pH throughout the operation. Two well‐defined reversible redox pairs are observed in PTO, each situated within the potential window bounded by the onset potentials of the HER and OER, when utilizing a cathode of platinum‐plated titanium mesh and an anode of RuO_2_/IrO_2_‐plated titanium mesh. The mediator exhibited exceptional activity in electrocatalytic, decoupled HER, and OER under strongly acidic conditions over repeated cycles, attaining Faradaic efficiencies approaching 100% for both OER and HER.

Wang et al. introduced PANI auxiliary electrodes in acidic media for decoupling OER from HER. A temporal gas separation was observed with this redox mediator during water electrolysis, promoting high operational flexibility. Functioning as a transitional charge storage medium, the electrode PANI exhibited a remarkable reversible discharge capacity of 125 mAh/g. Additionally, a method to regulate the equilibrium potential of the PANI electrode was established, allowing for the balancing of individual cell voltages. This control facilitated the sequential operation of the decoupled HER and OER at nearly identical electric potential of just 0.8 V [85]. The properties of various decoupling agents discussed in this review are summarized in Table 1.

**TABLE 1:** Some key parameters of the decoupling agents for water splitting.

Decoupling agents or redox mediators	Redox potential (V) versus RHE	Best‐reported Faradaic efficiency		References
		for HER, %	for OER, %	
Phosphomolybdic acid (PMA)	0.57 and 0.68	100	100	[28, 43, 56]
Silicotungstic acid (STA)	−0.20 and 0.04	95 ± 7	100	[51, 52, 56]
Phosphotungstic acid (PTA)	−0.04 and 0.24	44.6	—	[56]
Silicomolybdic acid (SMA)	0.51	—	—	[56]
Vanadium(III)/vanadium(II)	−0.26	96 ± 4	—	[62, 63]
Cerium(IV)/vanadium(III)	1.48	—	78 ± 8	[62]
Dihydroxyphenazinesulfonic acid (DHPS)	−0.05	100	100	[44]
Fe(CN)_6_^4−^/Fe(CN)_6_^3−^	1.19–1.37	99.88	—	[44, 64, 65]
Ferrocenylmethyl)trimethylammonium chloride (FcNCl)	1.01	100	100	[65]
Potassium hydroquinone sulfonate (PHQS)	0.65	98 ± 7	91 ± 5	[66]
Anthraquinonedisulfonic acid (AQDS)	0.214	100	100	[58]
Ni(OH)_2_/NiOOH	0.55	100	100	[25, 68, 77]
MnO_2_/MnOOH	0.15	~100	—	[86]
Ni–Mn–P	1.3–1.7	—	—	[80]
Polytriphenylamine (PTPAn)	0.7	97.8	—	[82]
Pyrene‐4,5,9,10‐tetraone (PTO)	0.7	98.7	96.5	[69]
Polyaniline (PANI)	0.8	—	—	[85]

Based on the discussion, it can be emphasized that soluble or solution‐dispersed redox mediators can freely circulate within the electrolyte. Their solubility allows rapid diffusion and effective charge transport, which can produce high current densities and facilitate fast reaction kinetics. Moreover, their redox potentials can often be chemically tuned, enabling them to fit in different reaction conditions, making them very attractive to scale the experimental design. The major disadvantage is, however, that they can contaminate the reaction products or lead to self‐discharge, while many redox couples suffer from chemical degradation during prolonged operation, which requires frequent replacement and complex recovery steps. On the other hand, solid redox mediators remain fixed on the electrode surface or within a confined layer. The mediator's immobility on the electrode surface significantly omits crossover and permits extended operational cycles. For compact electrolysis, solid mediators are highly advantageous. But, the electron transfer kinetics using solid mediators are often slower due to their less conducting nature and interfacial charge transfer, thus reducing the overall efficiency of the electrolyzer. Furthermore, the longevity of the solid redox mediators is significantly impacted by mechanical stress or phase transformations. Overall, for DWE to facilitate OER and HER, both solution‐dispersed and solid redox mediators provide unique pathways. Solution‐based redox mediators can promote rapid charge transport, but problem still exist in stability and product purity. The selection of an appropriate mediator is very critical for optimizing electrolyzer efficiency, stability, and scalability in DWE technology [55, 70, 87, 88]. Table 2 summarizes the various DWE systems with varyingoperational strategies, redox mediator types, operating conditions, FE, gas purity, scalability, and technology readiness level.

**TABLE 2:** Comparative benchmarking of representative DWE systems.

Entry	Redox mediator/auxiliary electrode	DWE strategy	Electrolyte	Current density, mA/cm^2^	Cell voltage	Faradaic efficiency	H_2_/O_2_ purity and gas crossover	Cycle/stability/durability	Scalability/system architecture	Cost and safety considerations	Technology readiness	References
1	Phosphomolybdic acid (PMA)	ECPB	1 M H_3_PO_4_	>100	2.94 V at 100 mA/cm^2^ current density	~100%	Complete temporal gas separation	500 cycles	Laboratory batch reactor	Polyoxometalate cost; acidic electrolyte handling	Proof‐of‐concept	[28]
2	Silicotungstic acid (STA)	Molecular metal oxide redox mediator	1 M H_3_PO_4_	>100	—	~100%	Complete gas separation	>40 h	Batch electrolysis	Polyoxometalate cost; acidic electrolyte handling	Proof‐of‐concept	[51]
3	V^3+^/V^2+^	RFB	1 M H_2_SO_4_	—	—	—	Complete gas separation	—	Flow battery configuration	acidic electrolyte handling	Pilot‐scale concept	[62]
4	Ni(OH)_2_/NiOOH	Solid‐state proton buffer‐mediated	1 M KOH	>100	1.6 V for HER and 0.4 V for OER at 200 mA current	94.7%	Overcomes gas crossover	100 cycles	Rechargeable system	Low‐cost Ni electrode	Laboratory scale	[68]
5	Polytriphenylamine (PTPAn)	Battery electrode‐mediated	0.5 M H_2_SO_4_	>100	1 V for H_2_ production process and 1.22 V for O_2_production process at 100 mA current	~100%	Complete gas separation	120 cycles	Rechargeable system	Organic polymer stability	Laboratory scale	[82]
6	Anthraquinone‐ 2,7‐disulfonic acid (AQDS)	ECPB	1 M H_2_SO_4_	>100	2.00 V at >3000 mA/cm^2^ current density	100%	Spatial gas separation	100 cycles	Hybrid electrolyzer	Organic mediator degradation	Laboratory scale	[58]
7	Cobalt additive incorporated NiOOH/Ni(OH)_2_	Electrochemical‐chemical cycle	5 M KOH	>100	1.44–1.60 V at 10–200 mA/cm^2^ current density	>99%	Spatial gas separation	100 cycles	Integrated reactor	—	proof‐of‐concept	[77]
8	Silicotungstic acid (STA)	ECPB in PEM cell	Electrolyte fluoride	>100	2 V at 500 mA/cm^2^ current density	99% decoupling efficiency	Membrane prevents gas crossover	200 h	PEM electrolyzer	Membrane cost	Laboratory scale	[52]
9	Bimetallic phosphide bifunctional catalyst (CoFeP) and activated carbon cloth as an auxiliary electrode	Supercapacitive membrane‐free electrolyzer	Tested with 1.0 M KOH and 0.5 M H_2_SO_4_	>100	1.76 V at 50 mA/cm^2^ current density	99%	Complete gas separation without membrane	100 cycles over 20 h operation	Membrane‐free integrated device	Low‐cost membrane less technology	Pilot‐scale concept	[23, 24]
10	Bi_2_O_3_/Bi^0^	Oxygen‐mediated DWE	1 M KOH	>100	∼0.45 V for HER and ∼1.19 V for OER at 5 mA current	100%	Temporal and spatial gas separation	>1000 cycles	Compact architecture	Membrane‐free cell	Laboratory scale	[38]
11	Nickel hydride battery	Battery‐assisted DWE	6 M KOH	>100	0.28 V for HER and 0.25 V for OER at 100 mA/cm^2^ current density	100%	Complete gas separation	35 cycles	Rechargeable integrated system	Commercial battery materials	Laboratory scale	[39]
12	Vanadium hexacyanoferrate	Liquid battery type redox mediator	6 M KOH	>100	1.0 V for HER and 0.84 V for OER at 100 mA current	97.8%	Complete gas separation without membrane	6000 cycles	Hybrid energy production	Hydrazine safety concern	Laboratory scale	[40]
13	[Fe(CN)_6_]^3−/4−^	Seawater direct electrolysis (SDE)	Alkaline seawater (1 M KOH + seawater	>100	~1.57 V at 100 mA/cm^2^	~85%	Suppressed chlorine evolution	5000 cycles	Scalable seawater electrolyzer	Cyanide‐complex handling	Pilot‐scale concept	[70]
14	Co–Ni(OH)_2_/CoNiOOH	ECPB	5.0 M KOH	<100	2.28 V at 50 mA/cm^2^	93.1%	Solid membrane acts as barrier for the separation of produced gases	>14 h	Electrolyzer device	Low‐cost nickel	Pilot‐scale concept	[89]
15	Silicotungstic acid (STA)	Solution‐phase redox mediator	0.5 M H_4_SiW_12_O_40_	>100	2.25 V at 100 mA/cm^2^	~100% decoupling efficiency	Complete gas separation	—	Flow‐cell electrolysis	Mediator recovery required	Laboratory scale	[55]

The long‐term performance of DWE is strongly governed by the stability, reversibility, and safety of the employed redox mediator. Solution‐phase mediators are susceptible to self‐discharge, crossover, irreversible redox reactions, and chemical decomposition, whereas solid‐state mediators generally undergo structural reconstruction, active‐material dissolution, phase transformation, and gradual capacity fading during repeated charge–discharge cycles [42, 68]. These degradation processes increase polarization, reduce the accessible redox capacity, and lower both Faradaic and energy efficiencies. Depending on the mediator chemistry, reported cycling stability ranges from ≈100–300 cycles for early POM‐based systems to more than 5000 cycles for Ni(OH)_2_/NiOOH and Prussian blue analogue mediators, while recently developed supercapacitive carbon electrodes have demonstrated stable operation exceeding 10,000 cycles with minimal capacitance loss [42, 68]. In addition to mediator degradation, several parasitic side reactions adversely affect DWE performance. These include mediator self‐discharge, hydrogen evolution at the mediator electrode, oxygen reduction by reduced mediator species, electrolyte decomposition, water oxidation or hydrogen evolution competing with mediator redox reactions, mediator over‐oxidation/over‐reduction leading to irreversible products, redox shuttle crossover between compartments, and corrosion or dissolution of active electrode materials [37]. These side reactions restrict hydrogen production, decrease electrolytic efficiency, and result in the mediator degradation. However, the environmental safety concern is in the priority in water electrolysis technology. The highly efficient redox mediator Br^−^/BrO_3_^−^ suffers from toxicity, volatility, and corrosiveness, which requires sealed systems along with corrosion‐resistant materials to operate the process [67]. Fe(CN)_6_^3−^/Fe(CN)_6_^4−^ couples possess excellent redox reversibility and relatively low toxicity under alkaline conditions; however, they may undergo photodecomposition or release toxic cyanide species under strongly acidic conditions [64]. Cerium(IV)/vanadium(III)‐based redox couples provide high redox capacities but require acidic electrolytes and raise concerns regarding metal dissolution, corrosion, and waste management [62, 63]. POMs exhibit excellent multielectron redox reversibility but are associated with relatively high material costs [60]. The organic quinone derivatives offer molecular tunability and lower environmental impact but may experience irreversible molecular degradation and limited long‐term cycling stability [58, 66]. Therefore, the development of next‐generation redox mediators should simultaneously maximize electrochemical reversibility, suppress parasitic side reactions, enhance long‐term durability, minimize toxicity and environmental impact, and reduce material costs to facilitate the practical commercialization of DWE technologies.

### 3.2 Catalysts in DWE

DWE has emerged as a compelling strategy to overcome the limitations of conventional electrolysis through the use of redox mediators and supercapacitive charge storage to decouple the electrochemical half‐reactions. The choice of appropriate catalysts is pivotal in DWE, as they facilitate mediator redox shuttling, efficient charge storage, or driving subreactions with minimal overpotential, producing high current densities (from a few hundred to >1000 mA/cm^2^) needed for industrial scalability. DWE decouples electrolysis through three main strategies: SRM‐based, SLRM‐based, and supercapacitive storage systems. SRM‐DWE, first demonstrated by Dotan et al., employed electrochemical‐thermally activated chemical (E‐TAC) approach and used platinized nickel‐coated stainless‐steel cathode and a Ni_0.9_Co_0.1_(OH)_2_ anode. Hydrogen evolution was carried out at ambient temperature with a current density of 50 mA/cm^2^. The applied cell voltage was restricted to remain within 120 mV above the thermoneutral voltage (1.48 V), minimizing heat loss and preventing undesired oxygen evolution or overcharging of the anode, which could induce the irreversible β‐to‐γ phase transition of NiOOH. The FE for the anode charging reaction was reported to reach 99.2% ± 0.1% of the applied charge [77]. Another example includes a Br^−^/BrO_3_^−^ shuttle in near‐neutral NaBr solutions, where Br^−^ is oxidized to BrO_3_^−^ at the anode during hydrogen evolution (Br^−^+3H_2_O → BrO_3_^−^ + 6H^+^ + 6e^−^), followed by catalytic decomposition in a separate reactor (2BrO_3_^−^ → 2Br^−^ + 3O_2_) using RuO_2_ catalysts. Platinum‐based cathodes (Pt/C) efficiently drive HER, while RuO_2_–TiO_2_/Ti dimensionally stable anode drives OER. This membraneless setup achieved efficiencies up to 97.6% higher heating value (HHV) of hydrogen at 50 mA/cm^2^ [67].

Platinum‐group metals (PGMs) like Pt/C remain benchmarks (*η* = 30 mV at 10 mA/cm^2^, Tafel slope of 30 mV/dec), but scarcity drives the search for non‐PGM alternatives. Transition metal carbides (e.g., V_8_C_7_ and Mo_2_C) can mimic platinum‐based materials and enable faster HER kinetics owing to the high‐density unsaturated metal sites. When these catalysts are integrated with decoupled setups, they can produce current densities of as much as 500 mA/cm^2^ in highly alkaline conditions [90].

In supercapacitive electrolyzers, CoFeP catalyst boosts active surface area, reducing ohmic drops to 100 mV during polarity switching. Ni_5_P_4_ and MoS_2_ further excel in flow‐cell‐decoupled systems, where high catalyst loadings mitigate mass transport limits. Recent advances include heterostructured phosphides (e.g., Ni_2_P/MoP) with interface synergies lowering Tafel slopes to 40 mV/dec. Chalcogenides like MoS_2_ influence basal/edge site engineering, where single‐atom catalysts (SACs) on N‐doped carbon (e.g., Ni‐SACs) achieve TOF = 12 s^−1^ by confining metals in single sites, preventing agglomeration [61, 91, 92]. The overview of different catalysts for DWE along with their key features is represented in Figure 6 [30, 37, 93, 94]. It can be summarized that noble metal‐based catalysts are highly efficient for HER/OER, but their high cost is the major limitation for their active participation in the electrocatalysis. Transition metal‐based electrocatalysts are good alternatives in terms of stability and catalytic performance.

**FIGURE 6:**
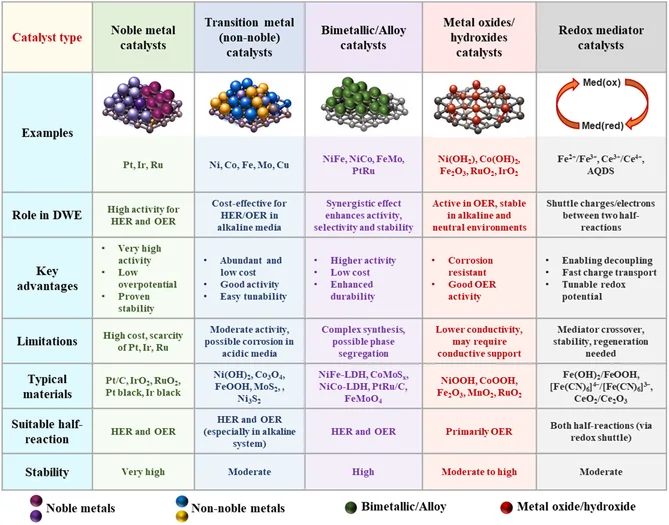
Overview of different catalysts for DWE.

### 3.3 Membranes in DWE

Various DWE systems are run with membraneless technology. In DWE, membranes are less commonly used than in conventional electrolyzers. The DWE setups employing membrane/without membrane are represented in Figure 7a–d. In redox‐mediated DWE shuttle species like Br^−^/BrO_3_^−^ or POMs (e.g., H_3_PMo_12_O_40_), membranes may separate anolyte/catholyte to retain soluble mediators, preventing crossover of the gases [70, 94]. For instance, in Ni(OH)_2_/NiOOH solid redox couples, no membrane is needed in batch mode, but continuous flow systems might employ porous diaphragms to minimize mixing of the gases while allowing OH^−^ flux [23, 24, 94]. The supercapacitive DWE electrolyzers use a single membrane‐free cell where an auxiliary electrode (e.g., activated carbon cloth) stores charge during HER, that discharges during OER, avoiding ion transport process during electrolysis [23, 24]. The bipolar solid‐state membrane, e.g., Co–Ni(OH)_2_/Co–NiOOH played a crucial role in DWE. The two surfaces of the solid‐state membrane acted like independent redox‐active interfaces to prevent produced gas cross‐over, while improving the safety and enabling the continuous gas production through polarity switching [89]. The bipolar solid membrane employed in the DWE cell is represented in Figure 7e. During the redox process, the solid membrane undergoes reversible transformation between Co–Ni(OH)_2_ and Co–NiOOH, facilitating the storage and release of electrons. Unlike conventional membranes, the solid mediator membrane can store and transfer the electrochemical charge between the two redox stages.

**FIGURE 7:**
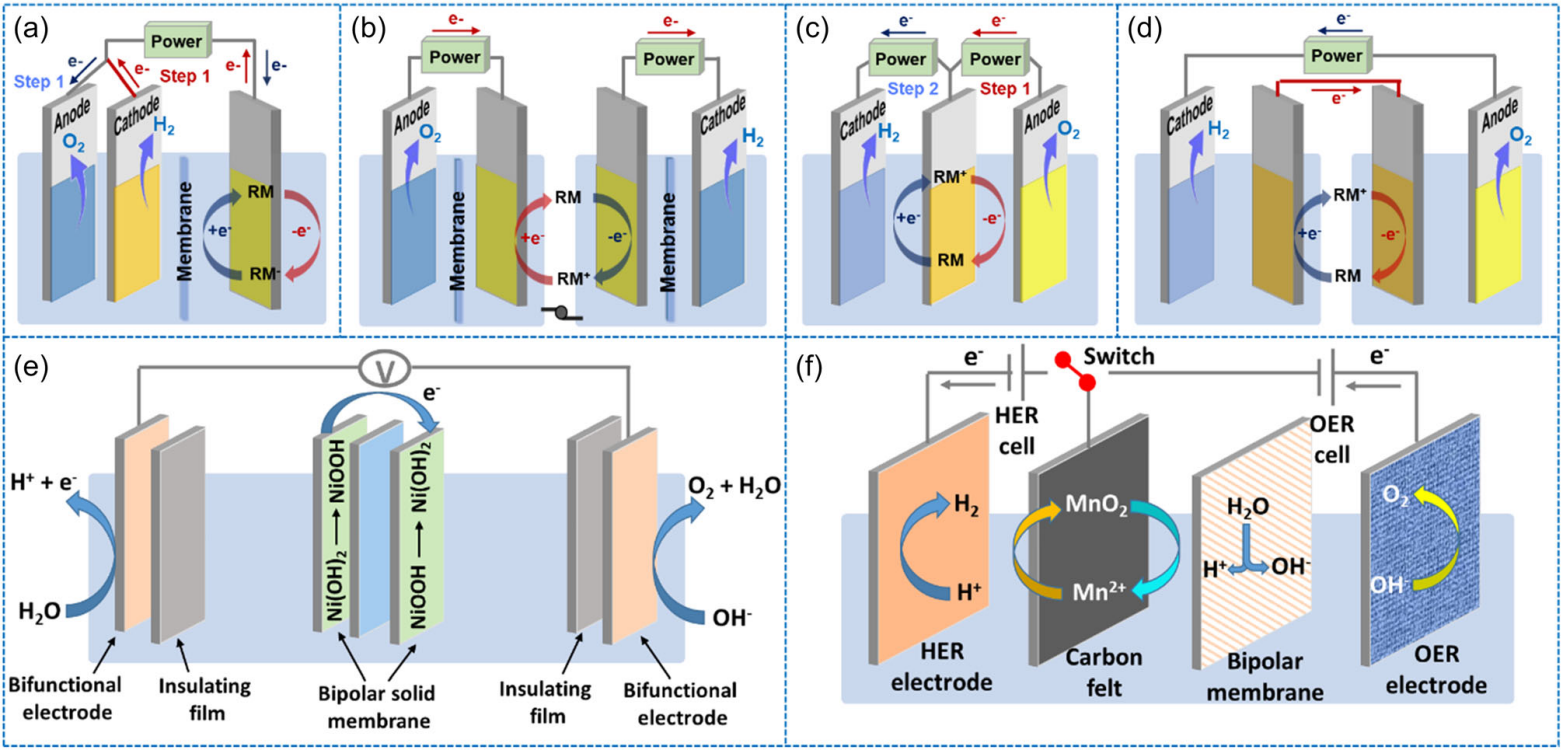
Various DWE cell configurations. (a) Two‐compartment configuration employing soluble redox mediator [95]. (b) Dual electrolyzer setup employing soluble redox mediator [95]. (c) Membrane‐free configuration employing solid redox mediator alternately mediating HER and OER [95]. (d) Closed bipolar electrochemical cell configuration employing solid redox mediator [95]. (e) Bipolar solid membrane assisted DWE cell configuration [89]. (f) Bipolar membrane assisted DAWE cell configuration [96].

Stage 1: HER and membrane charging occur following Equation (38).



(38)

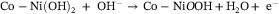




The solid redox mediator stores the released electrons, following the conversion of chemical state of solid membrane from a reduced state (Ni(OH)_2_) to an oxidized state (NiOOH) and enabling the HER at cathode (Equation (39)).

(39) $$2H_{2}\;O+2e^{-}\;\to \;H_{2}\;+2{\mathrm{OH}}^{-}$$

Stage 2: OER and membrane regeneration

Upon completion of the stage 1 process, the power polarity is reversed, reducing the membrane to Ni(OH)_2_ while facilitating OER as per Equations (40) and (41).

(40) $$Co‐NiOOH+H_{2}O\;+\;e^{‐}\to \;Co‐Ni{\left(OH\right)}_{2}+OH^{‐}$$

(41) $$4{\mathrm{OH}}^{-}\;\to \;O_{2}\;+2H_{2}\;O+4e^{-}$$

In the entire redox process, the solid membrane acts like an electron reservoir, allowing OER and HER to occur both at different times and space. This decoupled system is capable of performing continuous operation for more than 50 h, demonstrating its superiority over conventional membranes in AEM or PEM electrolyzers [89].

Decoupled amphoteric water electrolysis (DAWE) strategy is another advanced strategy that combines the advantages of amphoteric electrolysis with redox‐mediated decoupling for enhanced green hydrogen production. Huang et al. introduced the DAWE strategy with MnO_2_/Mn^2+^ redox mediator and bipolar membrane, enabling the temporal separation of HER and OER separation. The representative setup for DAWE is given in Figure 7f. However, despite several advantages, the bipolar membrane‐employed electrolyzer suffered from a serious drawback such as voltage drop across the membrane. The voltage drop originated from ohmic resistance associated with ion transport through the bipolar membrane and huge overpotential, arising from water dissociation at the junction of cation‐exchange membrane/anion‐exchange membrane in the bipolar membrane setup. The voltage drop was the main concern in the bipolar membrane‐assisted DAWE cell, for which current density obtained was substantially low (below 50 mA/cm^2^) and resulted in membrane delamination or swelling. However, employment of MnO_2_/Mn^2+^ redox mediator, the system became more efficient with enhanced operational stability and high current density of 1000 mA/cm^2^ [96].

Membrane‐free DWE highlights intrinsic mediator confinement or rapid kinetics, achieving >98% FE at 100 mA/cm^2^ [67]. Eliminating membranes reduces capital costs (20%–40% of stack prices in PEM) and enables dynamic operation with renewable energy sources. In a single electrolytic cell equipped with Br^−^/BrO_3_^−^ redox mediator could process HER upon oxidation of Br^−^ to BrO_3_^−^, and the process was followed by OER in a separate reactor. The process ensures the prevention of gas crossover through spatial separation. The process resulted in the continuous production of H_2_ and O_2_ at a cell voltage of ~1.8 V and generated 400 mA/cm^2^ current density. Another membrane‐free DWE technology equipped with CoFeP electrocatalyst delivered 26.6 mF/cm^2^ capacitance, with 20+ h operational durability at 100 mA/cm^2^ current density, showing 99% FE [23, 24, 55].

There are many DWE systems that use membranes for mediator stability. In POM (H_3_PMo_12_O_40_)‐enabled DWE system, spatial decoupling enabled production of gases with 95% round‐trip efficiency with higher energy cost. In diaphragm‐equipped dual cell employing NiOOH solid redox couple exhibited more 1000 h operational durability in highly alkaline medium. Quinone‐based redox mediator when combined with membranes allowed only ions to pass and thus prevented the mediator diffusion [89, 97]. Membrane less DWE technology showed current densities of 1000 mA/cm^2^ at lab scale, but industrial scale gas production with this process requires careful gas handling [55].

Membrane‐containing DWE systems use separators like AEMs or CEMs to confine redox mediators, generally exhibiting higher charge–transfer resistance (*R*_ct_) but improved safety compared to traditional PEM or AEM electrolyzers, where simultaneous HER/OER demands low *R*_ct_ ion conduction. In DWE, *R*_ct_ values range from 0.5 to 2 Ω cm^2^ for NiOOH‐mediated setups, elevated by mediator adsorption, which slows the kinetics, whereas, for PEM, *R*_ct_ values range from 0.1–0.3 Ω cm^2^ (for IrO_2_/Pt catalysts), and for AEM, it is 0.2–0.5 Ω cm^2^ (for NiFe/Cu catalysts) at 1000 mA/cm^2^. This is because DWE separates the reactions in time, which introduces interfacial polarization during charge shuttling [98]. In membrane‐bound DWE systems, mass transport losses are very low, around 10–20 mV at high currents of 1 A per square centimeter. This happens because DWE separates hydrogen and oxygen production into different steps, so gas bubbles don’t pile up on the electrode surfaces at the same time. Bubble coverage stays small, just 5%–10% of the electrode surface, compared to 20%–30% in regular systems (AEM and PEM), where both gases form together and block more spaces. Traditional PEM electrolyzers do little better, with losses under 5 mV where super‐thin Nafion membranes (20–50 µm‐thick) are used and lots of extra water is pumped in (over 50 times what's needed). Sometimes, the process is followed by drying of water from the flow channels, which causes a rise in temperature and deteriorates the electrolyzer's efficiency. The AEM electrolyzers have higher losses, 20–50 mV, because hydroxide ions (OH^−^) move slower through the liquid and the KOH electrolyte tends to swell and clog things up. DWE works well with near‐neutral chemicals like bromate (BrO_3_^−^), which let gases and ions to move easily through natural convection. This allows current densities above 2000 mA/cm^2^ without any blockages [55, 99]. Additionally, mass transport losses from concentration gradients and bubbles are markedly lower in DWE systems (10–30 mV at 1000 mA/cm^2^) compared to those in PEM and AEM electrolyzers (20–60 mV). Decoupling temporally separates gas evolution; HER bubbles during charging don’t overlap with OER, thus reducing coverage to <10% electrode area, but in PEM or AEM, the simultaneous gas bubbling clogs the pores of the electrodes. Moreover, reports suggest that at 2 V cell voltage, PEM electrolyzer leads (1000–3000 mA/cm^2^ industrially), followed by AEM electrolyzer (500–1500 mA/cm^2^), while membrane‐bound DWE reaches to 200–500 mA/cm^2^ [23, 24]. Durability favors PEM electrolyzer (>20,000 h at 1000 mA/cm^2^) and AEM electrolyzer (5000–10,000 h) over DWE (200–1000 h), limited by mediator degradation and parasitic side reactions. Ohmic resistance in DWE (0.1–0.3 Ω cm^2^ with diaphragms) rivals that of PEM (0.05–0.15 Ω cm^2^) but exceeds that of AEM (0.2–0.4 Ω cm^2^) due to thicker separators [98].

## 4 Multiscale Simulations for DWE

Computational electrochemistry has rapidly advanced with the development of constant‐potential/grand‐canonical density functional theory (DFT), enabling realistic simulations of electrochemical interfaces under experimentally relevant operating conditions. These approaches provide atomistic insights into electron transfer and interfacial charge redistribution, allowing the rational design of redox mediators and electrode architectures for DWE. Modern multiscale simulations integrate DFT, ab initio molecular dynamics (AIMD), continuum transport models, and machine learning (ML) to establish design principles for mediator selection and device optimization. Constant potential or grand canonical DFT is suitable for describing electrode reactions, whereas explicit solvent‐free energy calculations are required for molecular mediators whose redox thermodynamics are strongly influenced by hydration and hydrogen bonding interactions. For instance, high‐throughput DFT screening identified vanadium‐substituted Dawson POMs as promising redox mediators, demonstrating that the electronic structure and redox potentials govern multielectron storage and pH buffering during DWE operation (Figure 8) [100]. Mono‐vanadium‐substituted {P_2_W_17_V} exhibits reversible one‐electron redox activity with sequential proton‐electron transfer (SPET), whereas tri‐vanadium‐substituted {P_2_W_15_V_3_} undergoes reversible three‐electron redox reactions through cooperative proton‐electron transfer mechanism. Electrochemical measurements showed that {P_2_W_15_V_3_} operates within a suitable redox window (0.60 V vs. SHE) while maintaining rapid electron transfer kinetics (3.40 × 10^−2^ cm s^−1^), a diffusion coefficient of 2.36 × 10^−6^ cm^2^ s^−1^, and a Coulombic efficiency of 99.66%. Marcus theory and pKa calculations further demonstrated that V substitution lowers the proton‐transfer barrier, enhances proton coupling through the POMs framework, and improves the pH‐buffering capability during repeated redox cycling. Integration of {P_2_W_15_V_3_} mediator into a BiVO_4_‐based photocatalysis‐electrolysis relay system resulted in stable hydrogen production with a FE above 98.5%, demonstrating that computationally guided electronic structure engineering provides an effective strategy for developing high‐performance multielectron redox mediators for DWE systems. The performance of DWE also depends on the catalytic reaction pathway and the electrochemical regeneration of the redox mediator. In the ferricyanide/ferrocyanide ([Fe(CN)_6_]^3−^/[Fe(CN)_6_]^4−^)‐mediated decoupled seawater electrolysis system, anodic oxidation of [Fe(CN)_6_]^4−^ replaces the electrochemical OER, whereas [Fe(CN)_6_]^3−^ is chemically reduced back to [Fe(CN)_6_]^4−^ in a separate oxygen evolution reactor over an Fe–Ni(OH)_2_/NF catalyst to enable spatial separation of hydrogen and oxygen production [70]. The mediator exhibits quasireversible redox behavior in alkaline seawater with a redox potential of 1.27 V versus RHE, maintains less than 5% attenuation after 5000 cyclic voltammetry cycles, and displays diffusion‐controlled oxidation with a diffusion coefficient of 4.496 × 10^−6^ cm^2^ s^−1^ with an exchange current density of 0.201 mA cm^−2^. The integrated decoupled seawater electrolysis system operates at ~1.37 V at 10 mA cm^−2^ and ~1.57 V at 100 mA cm^−2^, while maintaining stable operation for over 250 h at 200 mA cm^−2^ without detectable chlorine‐containing byproducts even under chloride‐saturated conditions. DFT calculations demonstrated that adsorbed [Fe(CN)_6_]^3−^ modifies the OER pathway on the Fe–Ni (OH)_2_/NF catalyst by shifting the rate‐determining step from O → OOH** to OOH → O_2_* and reducing the corresponding free‐energy barrier from 0.44 to 0.36 eV, thereby facilitating oxygen evolution in the separate reactor and improving the overall efficiency of the decoupled seawater electrolysis process. The computed pathway is consistent with the high Faradaic efficiencies and low crossover observed when the ferricyanide redox couple is integrated with NiFe‐based anodes in alkaline saline media, thereby maintaining OER selectivity at practical current densities and suppressing chlorine oxidation and reduction [101].

**FIGURE 8:**
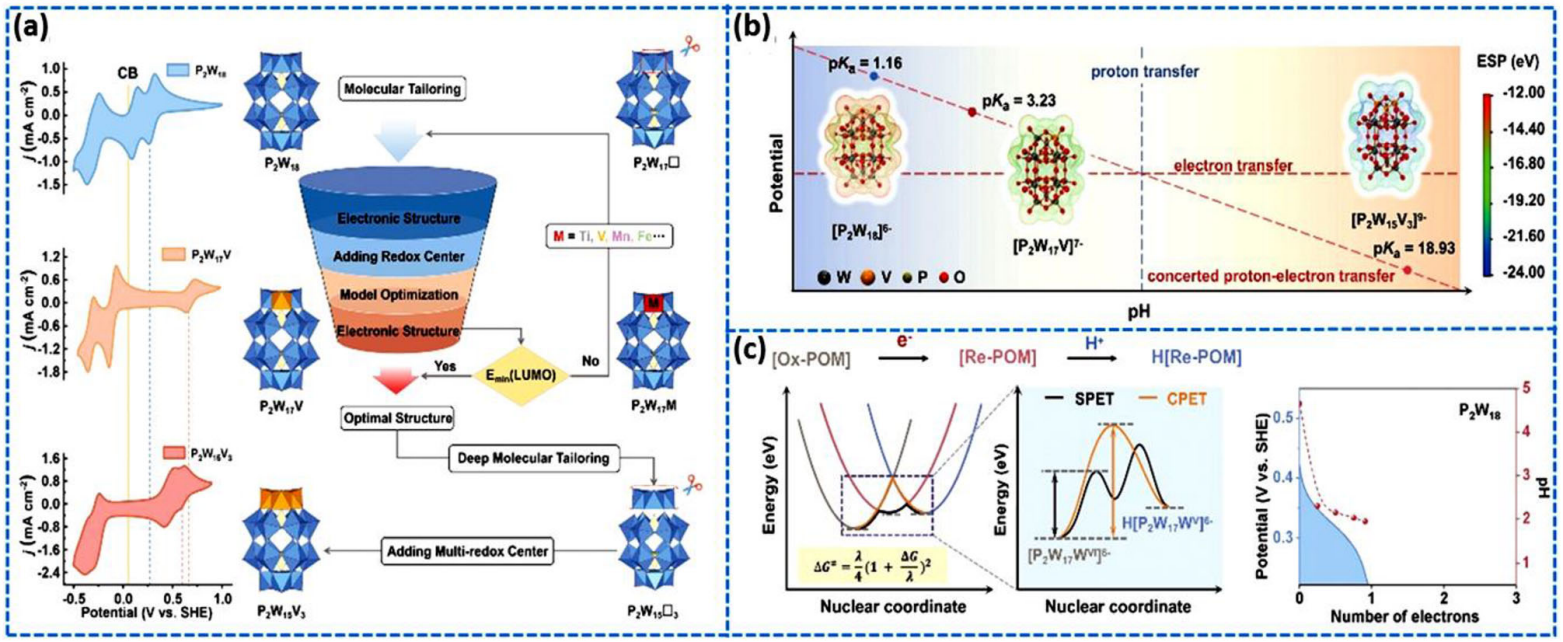
Multiscale simulations for DWE. (a) High‐throughput computational screening process of POMs‐based redox mediators. (b) pKa values and surface electrostatic potentials of the proton transfer products of (P_2_W_18_), (P_2_W_17_V), and (P_2_W_15_V_3_) redox pairs. (c) Marcus theory analysis of electron and proton transfer behaviors and the corresponding pH changes during (P_2_W_18_) electrolysis. Reproduced with permission [100]. Copyright 2025, Springer Nature.

For solid redox mediators, the key transport descriptor is the activation barrier for proton or ion migration through the host lattice, which governs ionic diffusivity and charge transport kinetics. In *α*‐MoO_3_, climbing image nudged elastic band (CI‐NEB) calculations revealed an anhydrous proton migration barrier of ≈0.13 eV, which is comparable to that reported for CuFe‐TBA (0.15 eV) and substantially lower than that of Ni(OH)_2_ (>0.6 eV) [81]. This low barrier arises from the dense oxide‐ion arrangement (O–O distance <3 Å), which supports proton hopping through a Grotthuss‐type mechanism. The favorable proton transport pathway is consistent with the high‐rate electrochemical response of *α*‐MoO_3_, which delivered a capacity of 204 mAh g^−1^ and retained 77% of its low‐rate capacity at 100 A g^−1^. In the decoupled electrolyzer, hydrogen production was sustained at current densities up to 10 A cm^−1^ with Faradaic efficiencies above 95%, demonstrating that rapid proton transport within the mediator translates into high‐rate hydrogen evolution. In situ X‐ray diffraction further revealed reversible lattice evolution with negligible interlayer expansion during proton insertion and extraction, confirming that proton transport proceeds with minimal structural perturbation. These results demonstrate that a low proton migration barrier supports rapid mediator deprotonation and high‐rate hydrogen production in DWE. Microkinetic and transport models can incorporate DFT‐derived migration barriers into continuum simulations to predict polarization behaviors and energy losses under dynamic operating conditions [102]. These continuum framework map onto intermittent‐power cycling protocols, thereby predicting the effect of mediator diffusion, buffer state‐of‐charge, and electrode porosity on the dynamic cell voltage during step‐switching [103]. For organic electron‐coupled proton buffers (ECPBs), the electrochemical behaviour of mediators can be evaluated through Pourbaix diagrams, which establish the thermodynamically stable oxidation and protonation states over the pH range. Gaudin et al. developed a first‐principles DFT/COSMO‐RS workflow that employs Nernst and Henderson–Hasselbalch equations to predict pH‐dependent redox behaviour of quinone‐based mediators [104]. This approach reproduced experimental Pourbaix diagrams of four anthraquinone derivatives with an RMSE of 0.9 pKa units (*R* ^2^ = 0.89) for acid dissociation constants and RMSE of 0.052 eV for standard reduction potentials over a range of −0.1 to 0.9 V versus SHE. The predicted diagrams accurately captured pH‐dependent changes in reduction potential, protonation equilibria, and slope transitions arising from multiple proton‐coupled electron transfer processes. The study demonstrated that the empirical COSMO‐RS parameterization for pKa calculations effectively captures hydration effects that previously required computationally expensive QM/MM calculations, thereby significantly improving the prediction of outlier anthraquinones. These approaches provide an efficient computational strategy for screening organic redox mediators with suitable potential‐pH operating range, proton‐buffering capability, and reversible PCET behaviour, which are essential characteristics for high‐performance DWE systems. Although Pourbaix diagrams identify thermodynamically preferred redox and protonation states, they do not capture kinetically accessible reactions such as nucleophilic water addition, radical dimerization, or irreversible bond cleavage, indicating that aqueous stability must be evaluated from explicit decomposition reactions. The corresponding reaction‐free energies should be calculated for all relevant oxidation and protonation states, followed by transition state calculations for pathways that are thermodynamically accessible.

Frustrated Lewis pairs (FLP)‐coated NiFe‐LDH operates as an engineered interface that optimizes solvation and electronic structure. Zhu et al. employed AIMD simulations on CeO_2_ layers with spatially separated Lewis acid/base sites and found hydroxide accumulation closer to the surface when compared to that with chloride, enabling selective seawater oxidation [105]. DFT confirms this selectivity through stronger OH adsorption and weaker Cl binding on NiFeLDH coated by oxygen‐vacancy‐derived FLP model. Free energy analysis on Ni–Fe oxyhydroxide models identifies nickel oxyhydroxide as an active phase with a lower energy barrier of 0.50 eV at 1.23 V, shifting the rate‐determining step from OH adsorption in the non FLP model to the *O → *OOH step in the FLP model. Projected density of states reveals gradient coupling in which Ce 4*f* donates electron density through O 2*p* into Ni 3*d*, creating oxygen nonbonding states that stabilize the Ni–O bond. The electronic modulation pushes Δ*G*(*OOH) ‐ Δ*G*(OH) from the canonical ~3.2 eV toward the ideal 2.46 eV, weakening the usual scaling constraint and lifting intrinsic OER activity in alkaline seawater. The outcome aligns with alkaline‐seawater demonstrations in which mediator‐assisted anodes suppress chlorine evolution and maintain OER selectivity at fixed current density. However, conventional AIMD trajectories generally sample picoseconds to nanoseconds, whereas mediator decomposition and electrode reconstruction develop over hours to thousands of cycles. Continuum models can translate calculated diffusivities and heterogeneous rate constants into mediator concentration profiles and crossover fluxes, but these parameters require experimental validation. ML screening is most reliable for molecular quantities that can be generated by quantum chemistry, including redox potential, pKa, solvation‐free energy, and frontier orbital‐based reactivity. These models down‐select organic mediators with appropriate PCET symmetry and low membrane sorption, leading to a shift toward higher‐capacity tungstates and the selection of AQDS over PHQS for durable cycling. Mao et al. developed a high‐throughput screening over 7924 Cu‐based alloy nanoclusters to identify stable and active HER electrodes [106]. This approach established a surface charge‐difference descriptor that correlates linearly with the count of optimal sites, providing a tool for charge–transfer prediction. A neural network trained on DFT dataset achieved a mean absolute error of ~0.07 eV in predicting Δ*G*_H*_, allowing fast screening of material compositions. The core–shell configurations with transition‐metal cores and Cu shells exhibit superior catalytic performance due to optimized charge transfer and surface activity. Particularly, CuNi alloy clusters demonstrated the highest structural stability and HER activity with active sites localized on Cu bridge and three‐fold surface atoms. Transition‐metal and nonmetal‐doped 2D graphyne‐based SACs (TM‐NM‐GY) demonstrate that key features such as charge transfer, metal binding height, d‐band center, bond length of the coordinating atoms, and integral COHP (ICOHP) govern HER activity [107]. Enhanced electron donation from the metal site to hydrogen significantly optimizes Δ*G*_H*_, resulting in superior catalytic performance. The ML stacking ensemble model achieved better predictive accuracy (*R* ^2^ = 0.87), effectively capturing the relationship between the TM coordination environment and electron donation to adsorbed hydrogen. For DWE systems, this approach supports rapid co‐optimization of HER electrodes and mediator environments by targeting surfaces that maximize exchange current at operating potential, minimize two‐step voltage penalties, and sustain stable charge transfer under realistic conditions. Jinnouchi et al. introduced a ML‐aided first‐principles framework that combines thermodynamic integration with Δ‐ML corrections to optimize free energies from semilocal to hybrid functional [108]. ML force fields generate extensive ensembles, and alignment of redox states on the absolute potential scale through the O 1*s* reference yields aqueous redox potentials for Fe^3+^/Fe^2+^, Cu^2+^/Cu^+^, and Ag^2+^/Ag^+^, matching experimental values with near ab initio accuracy for solution‐phase redox thermodynamics. For solid buffers and electrochemical interfaces, *E*(3)‐equivariant ML interatomic potentials (NequIP and MACE) reach meV Å^−1^ force accuracy with orders‐of‐magnitude fewer ab initio reference calculations than conventional approaches [109]. This data efficiency enables long‐time large‐system molecular dynamics to resolve dissolution, lattice oxygen activation, and mediator‐induced corrosion. The tensor‐equivariant message passing preserves energy conservation and reproduces reactive dynamics. These advances elucidate the fundamental behavior of redox mediators and solid buffers, providing design principles that align with experimental progress in device architectures to accelerate the development of safe, flexible, and scalable decoupled electrolyzers for green hydrogen production.

## 5 Electrolyzers’ Techno‐Economic Analysis

The cost distribution of electrolyzer stacks varies considerably among different electrolyzers due to differences in materials, operating conditions, and technological maturity, as represented in Figure 9. The principal cost components include the HER catalyst, OER catalyst, membranes, bipolar plates (BPs), and gas diffusion layer (GDL) in AWE, AEM, and PEM technologies. Although AWE systems generally exhibit the lowest stack manufacturing cost because of their simple design and inexpensive catalysts, their comparatively lower current and power densities increase the cost of hydrogen production. Conversely, AEM or PEM electrolyzers possess higher stack manufacturing costs due to their expensive membranes or catalysts but can achieve lower hydrogen production costs owing to their superior electrochemical performance and efficiency. The contribution of the HER catalyst to total stack cost differs significantly across electrolyzer technologies. In PEM electrolyzers, the HER catalyst accounts for only 4.4% of the total stack cost, whereas it is ≈16.7% for AEM electrolyzers. However, the PEM electrolyzers possess the largest OER catalyst‐related expense, which is ≈32.3% of stack cost, unlike the lower stack cost associated to the AWE (9.8%) and AEM (2.7%) systems. Because OER is kinetically sluggish than HER, it requires highly active catalysts to reduce overpotentials. Membrane cost in AEM electrolyzers accounts for almost 60% of the total stack cost. This high cost is mainly attributed to the limited commercial availability and complex fabrication of highly conductive, chemically stable anion‐exchange membranes. However, as membrane synthesis techniques mature and manufacturing scales increase, significant cost reductions can be anticipated. Membranes contribute ≈11.4% and 28.3% of the stack cost in AWE and PEM electrolyzers, respectively. BPs also represent an important cost component, contributing ≈26.6%, 6.1%, and 11.7% of the stack cost in AWE, AEM, and PEM electrolyzers, respectively. Material selection is strongly influenced by the corrosive operating environment, requiring higher corrosion resistance in acidic PEM systems. The GDL contributes most significantly to AWE stack cost (52.2%). In PEM electrolyzers, the GDL, together with the catalyst layer and ionomer, contributes ≈23.3% of total stack cost due to the use of costly carbon–fiber‐based porous transport materials. Beyond stack components, system‐level costs indicate different priorities for future technological improvements. For AWE, reductions in stack or balance‐of‐plant (BoP) costs offer limited benefits because these costs are relatively low. Instead of improving energy efficiency by minimizing electrode overpotentials and reducing electricity consumption, it represents the most effective strategy for lowering hydrogen production costs. AEM systems would benefit primarily from reductions in membrane, catalyst, GDL, and BoP costs, alongside enhanced catalyst activity to decrease operational energy losses. In PEM electrolyzers, substantial cost reductions depend on decreasing the expense of BoP components, developing standardized large‐scale manufacturing processes, and replacing the precious metal catalysts with low‐cost alternatives. Future technological progress is expected to improve the economic competitiveness of all electrolyzer technologies, particularly through longer stack lifetimes. As operational lifetime increases, the capital cost associated with stack replacement is distributed over greater hydrogen production, thereby reducing the levelized cost of hydrogen. AWEtechnology currently provides the lowest hydrogen production cost because of its low initial capital investment. Recently, AEM and PEM technologies have evolved with reduction in cost owing to the advanced catalyst design, durable membrane, advanced manufacturing process, and optimization in the electrolyzer's operation. The future projections of conventional electrolyzers indicate that the hydrogen generation cost can decrease from 25.4 to 17.9 RMB/kg for the AEM technology and 39.9–19.6 RMB/kg for PEM technology. The component‐wise cost breakdown of conventional electrolyzers for green hydrogen generation, along with projected cost reductions under short‐, medium‐, and long‐term technology development scenario, are represented in Figure 9a–i [110].

**FIGURE 9:**
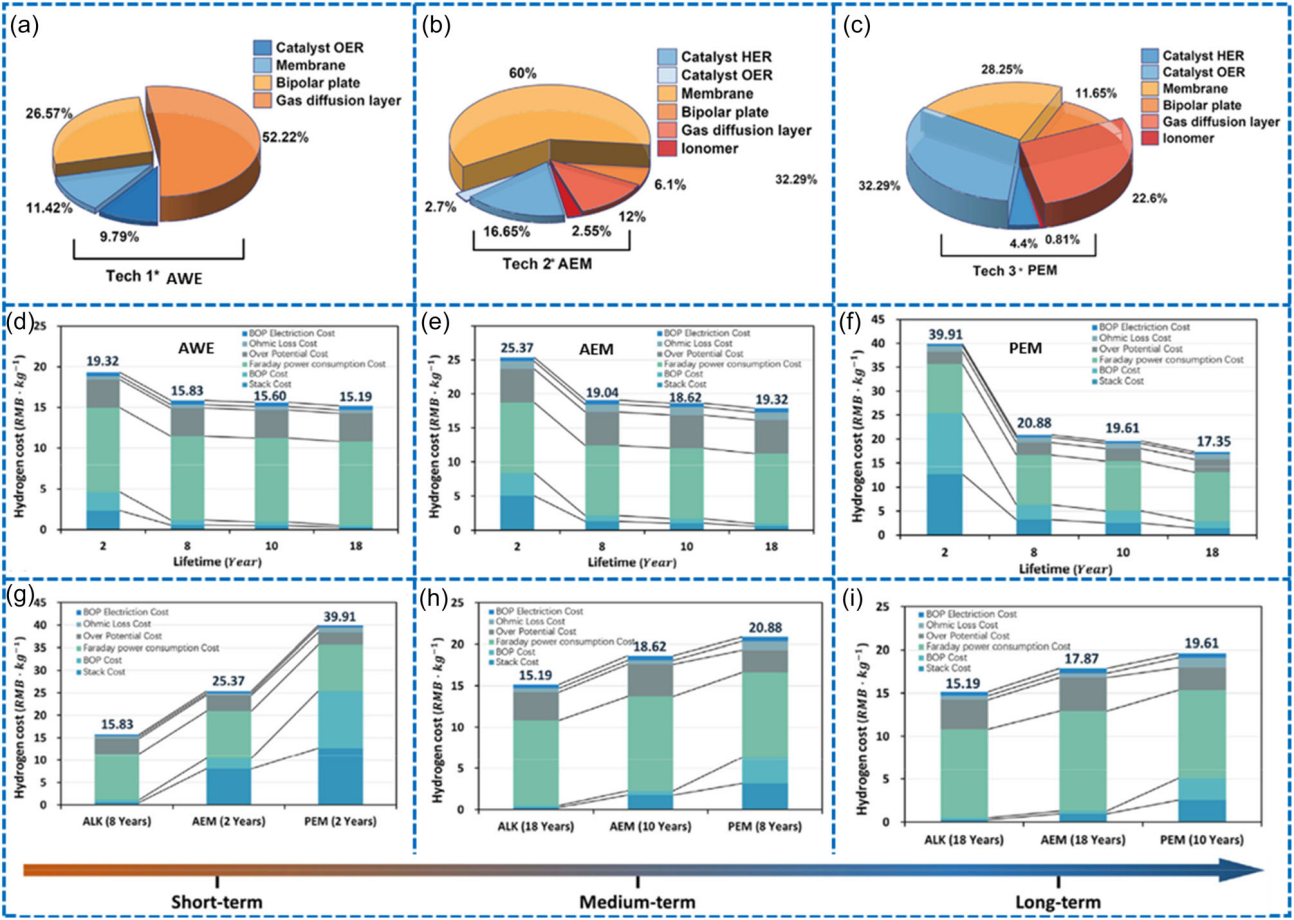
Cost analysis of different conventional electrolyzer technologies. (a–c) Component‐wise stack cost distribution for HER and OER catalysts, membrane, BPs, GDL, and ionomer, for (a) AWE, (b) AEM, and (c) PEM electrolyzers. (d–f) Hydrogen production cost as a function of 2, 8, 10, and 18 years stack lifetime for (d) AWE, (e) AEM, and (f) PEM electrolyzers. (g–i) Projected hydrogen production costs for (g) short‐term, (h) medium‐term, and (i) long‐term technology development scenarios. Reproduced with permission [110]. Copyright 2023, Elsevier.

The manufacturing cost of SOEC technology for electrolyte‐supported cells (ESCs) depends on the cost of raw materials, cost of processing, and operation. A detailed cost analysis reveals that the 10 mol% scandia‐stabilized zirconia (10‐SSZ) electrolyte contributes the major expense, which can range from US$350 to US$480/kg. However, other electrolyte such as nickel oxide (NiO) can decrease the overall stack substantially with increase manufacturing volume, compared to other electrolytes such as GDC and lanthanum strontium cobalt ferrite (LSCF). The raw material cost of ESC stack can be significantly reduced to 50% from ≈US$104/kWe DC_SIP_ to US$51/kWe DC_SIP_ (direct current system input power, DC_SIP_) upon increasing the manufacturing capacity from 25 MWe to 2000 MWe (Figure 10a). Among the various cell components in SOEC, the 10‐SSZ electrolyte demands major cost, but the stack cost can be significantly reduced from US$26.04 to US$11.86/kWe upon increase in manufacturing volume. The labor cost can be significantly reduced from US$18/kWe DC_SIP_ to US$8/kWe DC_SIP_ upon automated production process for high volume production. With increase in production capacity, the manufacturing and tooling costs can be declined. The manufacturing cost deceases from US$207 to US$37/kWe upon lab‐scale to industrial‐scale production, which also aligns with tooling cost from US$24 to US$10/kWe in the same production range. The major significant cost contributor is the high‐temperature heat treatment process. Other expenses include cell sintering, coating firing, and glass seal curing, which collectively demand 38%–55% of total manufacturing and tooling expenses (Figure 10b,c). The manufacturing cost of hydrogen electrode‐supported cells (HSCs) rely on selection of constituent materials, cell architecture, fabrication methodology, and production scale. But, the significant structural modification in HSCs compared to ESCs is the replacement of 10‐SSZ electrolyte with the more economical 8‐YSZ electrolyte, which causes the overall cost reduction in HSCs technology over ESCs technology. But, the compositions of GDC barrier layer and LSCF oxygen electrode remain fixed ESCs and HSCs configurations. However, the barrier layer thickness in HSCs is reduced to ≈5 mm to lower the material cost (Figure 10d) [111].

**FIGURE 10:**
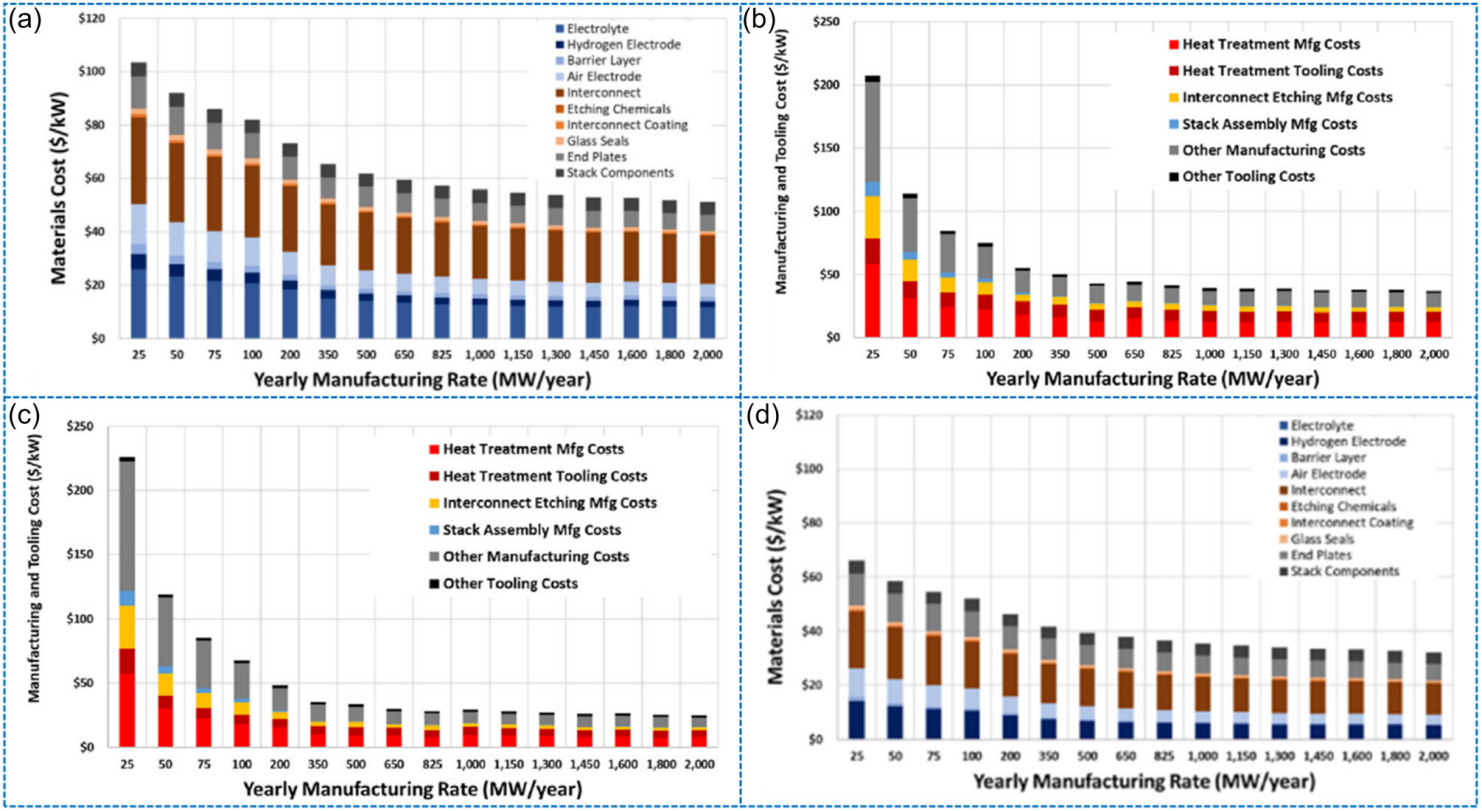
SOEC's cost analysis. (a) ESC stack versus HSC stack for materials cost. (b) ESC stack versus HSC stack for manufacturing and tooling costs. (c) ESC stack for manufacturing and tooling costs in 25–2000 MWe DC_SIP_ manufacturing rates range. (d) ESC stack for materials costs in 25–2000 MWe DC_SIP_ manufacturing rates range. Reproduced with permission [111]. Copyright 2024, Elsevier.

Other than stack manufacturing, SOEC systems are also associated with BoP, comprising compressors, heat exchangers, power electronics, piping, and auxiliary unit costs. If the annual production capacity is 1000 MWe DC_SIP_, BoP components remain the dominant contributors to facility cost. The direct capital cost and total capital investment can be expected asUS$931/kWe DC_SIP_ and US$1,134/kWe DC_SIP_, respectively, for 1 GWe DC_SIP_ SOEC plant, as presented in Figure 11a. Thermal energy required for steam generation is ≈US$0.33/kgH_2_, although this cost could be reduced using low‐cost heat sources such as nuclear energy. The analysis indicates that LCOH reductions become marginal beyond 500 MWe DC_SIP_, and achieving the DOE target of US$2/kg H_2_ requires electricity prices of US$0.020–0.025/kWh or lower under high plant utilization. The LCOF for various SOE facility is represented in Figure 11b–d [111].

**FIGURE 11:**
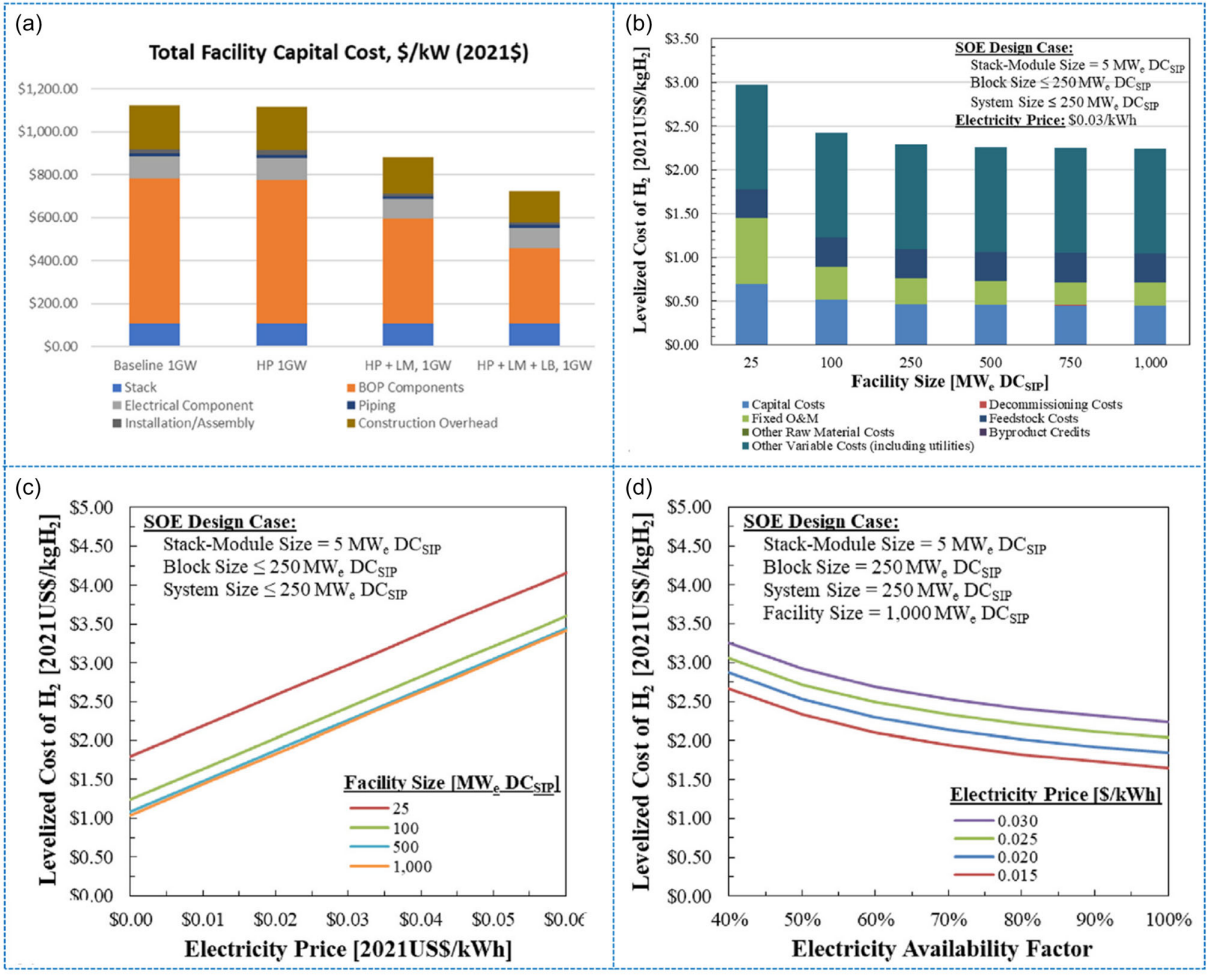
SOEC's cost analysis. (a) Total facility cost by major category for v1 GWe DC_SIP_ SOE facility. (b) LCOH breakdown for SOE facilities ranging from 25 to 1000 MWe DC_SIP_ under cumulative cost‐reduced design configuration. (c) Effect of electricity price on LCOH for facilities of different capacities. (d) Influence of electricity price and availability factor LCOH for 1 GWe DC_SIP_ SOE facility, a constant thermal energy demand of approximately 7.93 kWh_t_/kgH_2_. Reproduced with permission [111]. Copyright 2024, Elsevier.

There are very limited techno‐economic data available for DWE systems because most DWE technologies remain at the laboratory or pilot scale. Therefore, the techno‐economic analysis of DWE technology relies on different assumptions, and the parameters are not directly comparable. However, a few companies have started commercializing this DWE technology for green hydrogen production, demonstrating its potential to reduce capital expenditure, improve energy efficiency, and lower LCOH through simplified system architecture and membrane‐free operation [112, 113, 114, 115, 116]. For any electrolyzer, experimentally measured performance, such as cell voltage, current density, efficiency, and stability, can be analyzed separately from modeled cost estimates and industry targets. For a proper techno‐economic evaluation, factors such as stack lifetime, capacity factor, electricity price, BoP costs, mediator consumption and replacement, maintenance, water treatment, and additional energy requirements should also be considered along with the stack cost. This is especially important for DWE because temporal/spatial gas separation technology can reduce membrane‐related costs, but other additional costs related to redox mediators, fluid handling, control systems, and multiple or alternating electrochemical units may also be required. Therefore, the DWE's techno‐economic analyses including CAPEX and LCOH should be considered as specific targets unless they are confirmed by self‐reliant, system‐level studies. Previous techno‐economic studies of conventional AWE and SOEC have shown that LCOH strongly relies on plant configuration, electricity price, operating conditions, capital cost, and future technology improvements. The economic potential of DWE should be evaluated using similar system boundaries rather than by comparing individual stack‐level performance or projected costs. At present, DWE can be considered a promising technology with potential economic benefits; however, long‐term pilot‐scale operation and standardized techno‐economic studies are still required to confirm its competitiveness with already established AWE, PEM, AEM, and SOEC technologies.

## 6 Critical Perspective

The review's critical perspective involves not only summarizing the previously available reports of DWE technology but also evaluating its real‐world implications, limitations, and long‐term significance. While recent developments in device architectures such as redox mediator‐assisted systems and hybrid photoelectrochemical designs have significantly improved energy efficiency, operational stability, and material utilization, many of these strategies remain confined to laboratory‐scale demonstrations. Challenges, including mediator degradation, material cost, complex fabrication, and scaling constraints, continue to hinder real‐world deployment. Despite significant progress in photoelectrochemical water splitting including (STH) efficiency up to 15%–20% and stability beyond 120 h, key challenges remain before industrial implementation can be realized. Current high‐performance devices often rely on moisture‐sensitive absorbers, expensive materials, and complex system designs that increase capital and operational costs. While pilot‐scale demonstrations have established the technical feasibility of solar‐driven hydrogen production, achieving long‐term durability and cost‐effective scalability remains essential. Nevertheless, substantial challenges remain in translating laboratory‐scale performance into real‐world operation, particularly under fluctuating renewable energy inputs and prolonged outdoor exposure. More rigorous techno‐economic evaluation and long‐term pilot studies are therefore essential to assess system viability under practical operating conditions. A critical review can help identify existing knowledge gaps and highlight pathways toward the development of reliable, scalable, and commercially deployable green hydrogen technologies.

## 7 Uniqueness of This Review

This review demonstrates the critical analysis of DWE technology and its commercial roll‐out for mitigating the limitations of traditional electrolyzer technologies. The previously reported articles on DWE technology have summarized either the redox mediators or electrocatalysts used or operational strategy, but this review highlights mediator chemistry, comparing the membrane technology, catalysts families, reactor configurations, techno‐economic analysis, as well as discusses about technology readiness level of the DWE systems. The review further highlights the in silico mechanistic insights to understand the structure–property–performance relationships in atomic scale for designing the next generation DWE systems. It also compares the DWE technology to conventional electrolyzer technology in terms of safety improvement, operational flexibility, operational efficiency, and cost economy. This combined approach not only provides a broad conceptual knowledge but also delivers targeted, mechanistic insights, which can render the review a valuable resource for advancing green hydrogen production technologies for industrial implementation.

## 8 Gaps and Challenges

DWE is considered to be the next‐generation technology for green hydrogen production. It can produce H_2_ and O_2_ in independent chambers and/or at different times, which is impossible in conventional electrolysis. The production of H_2_ and O_2_ in different chambers can help mitigate the product gas purification process, ensure safety, and integrate better with intermittent power supply. Despite several advantages, DWE also faces several challenges for its potential commercialization. The key challenge is the use of cost‐effective and durable redox mediators or electrocatalysts for efficient production of clean energy. Other significant hurdles of DWE are material corrosion and membrane degradation, basically in harsh environmental conditions, which can severely affect the system's reliability. The DWE systems also need to be highly compatible to integrate with intermittent renewable power supply. Beyond the technical issues, the lack of standard operating protocols further limit commercial deployment. For the continuous hydrogen production, DWE requires powerful monitoring systems which can regulate the power supply, mediator concentration, system pressure, pH, and electrolyte flow. In addition, integration with intermittent energy supply needs huge attention for the adjustment of charge–discharge rates to prevent the system disintegration. Proportional‐integral‐derivative (PID) controllers are the industry standard but face significant limitations in managing electrolyzer stacks powered by fluctuating renewable energy sources. Since in DWE the reactions are decoupled, rigorous balancing of gas pressures and membrane cross‐over is simplified, reducing the reliance on complex, high‐frequency PID tuning, allowing a more robust, efficient operation even under variable loads. Moreover, in silico study can show promises to meet the challenges, but the lack of vast dataset limits the applications of DWE in industrial scale.

## 9 Prospect and Future Outlook

DWE technology is innovative for green hydrogen production due to its ability to spatially and temporally separate the HER from OER processes. Despite major advantages, DWE also faces several challenges related to its economic feasibility, stability, and adaptability. Research into organic molecules, metal complexes, and solid‐state mediators is ongoing, but scalability and stability remain challenging. Equally important are advanced electrode materials and catalysts such as metal–organic frameworks, conductive polymers, and transition‐metal oxides that can sustain efficiency under decoupled operation. Thermal management as well as energy recovery strategies are very crucial for DWE technology to make it more efficient, durable, and commercially viable. As decoupled electrolyzers can easily integrate with renewable energy, it is positive, but intermittent power can lead to uneven heat distribution throughout the system, challenging the stability of redox mediators and durability of the components as well as the system efficiency. The uneven heat distribution can be minimized with incorporation of phase‐change materials or microstructured heat exchanger or thermally conductive materials, such as introduction of graphene‐based pipelines or thermal insulators, which can help in the energy recovery process too. Moreover, with various R & D investments, pilot projects can boost the DWE technology to become a keystone infrastructure in safe, flexible, and sustainable green hydrogen production.

## 10 Industrial Roadmap for Next Decade

Conventional water electrolysis technology offers high technology readiness level (TRL 9); on the other hand, the DWE technology remains in early lab scale stage development (TRL ≤ 5). The SwedishCaplyzer technology based on supercapacitive DWE is at TRL 6 [112]. The E‐TAC process, developed by H_2_Pro represents DWE approach based on sequential electrochemical and chemical steps (TRL 5) [113]. Similarly, Clyde Hydrogen has demonstrated DWE prototype and is capable of producing green hydrogen from an intermittent renewable power supply [114]. UK‐based industry CPH_2_ has developed membrane‐based hydrogen production approach, facilitated by cryogenic downstream gas separation technology [115]. On the other hand, sHYp's membraneless technology can produce green hydrogen from seawater without the desalination process [116]. Hydrox Holdings’ developed divergent‐electrode‐flow‐through alkaline approach using porous electrodes at elevated temperatures (200–300 °C) [117], and Supercritical Solutions Ltd.'s high‐temperature/high‐pressure system achieves 99% HHV efficiency while bypassing compression, aiming for hydrogen costs below £1/kg by 2030 [118]. Additionally, supercritical water oxidation of organic and biomass feedstocks offers a nontraditional but promising pathway, generating hydrogen‐rich gas via partial oxidation and subsequent water–gas shift processing under supercritical conditions (>374 °C, >22 MPa) [119, 120, 121].

Although the decoupled pilot electrolyzers are deployed by many industries, still they struggle to demonstrate high operational durability because of the mediator/electrocatalyst degradation, utilization of intermittent renewable power inputs, etc. These limitations can be mitigated by the application of stable redox mediators (the conductive polymers/metal oxides‐based redox mediators can be preferred) which can be capable of minimize mediator loss, parasitic side reactions, and gas crossover. As DWE is capable of separating the HER and OER either at different times or space, hence, modular architectures in the advanced reactor and component design can be helpful to operate the two reactor units independently under different operating conditions or chemical environment, which can limit the mechanical stress, catalyst dissolution, or corrosion effect in the independent unit. Additionally, controlled ON/OFF process can minimize the transient voltage spikes and abrupt power changes. Periodic regeneration cycles such as controlled polarity reversal (reversal of current direction to remove insulating oxide layers or other impurities deposited on the electrode surface), electrolyte conditioning (partial replacement or purification of electrolyte to remove contaminants), redox rebalancing (controlled and periodical charging/discharging protocols can be applied to the redox mediator to minimize the drifting in optimal oxidation states due to parasitic side reactions or incomplete redox process), and potential/current hold in the specific value (holding the system at fixed potential or current can enable the removal of impurities to allow the catalysts to retain in their favorable oxidation states). Recently, H_2_Pro is trying to adopt these strategies to hit the target of achieving high operational durability with efficient hydrogen production by 2035. But, bridging these technologies from pilot‐scale demonstration to industrial deployment necessitates targeted scale‐up strategies, robust techno‐economic analysis, and staged TRL advancement, as reflected in the proposed commercialization roadmap (Figure 12).

**FIGURE 12:**
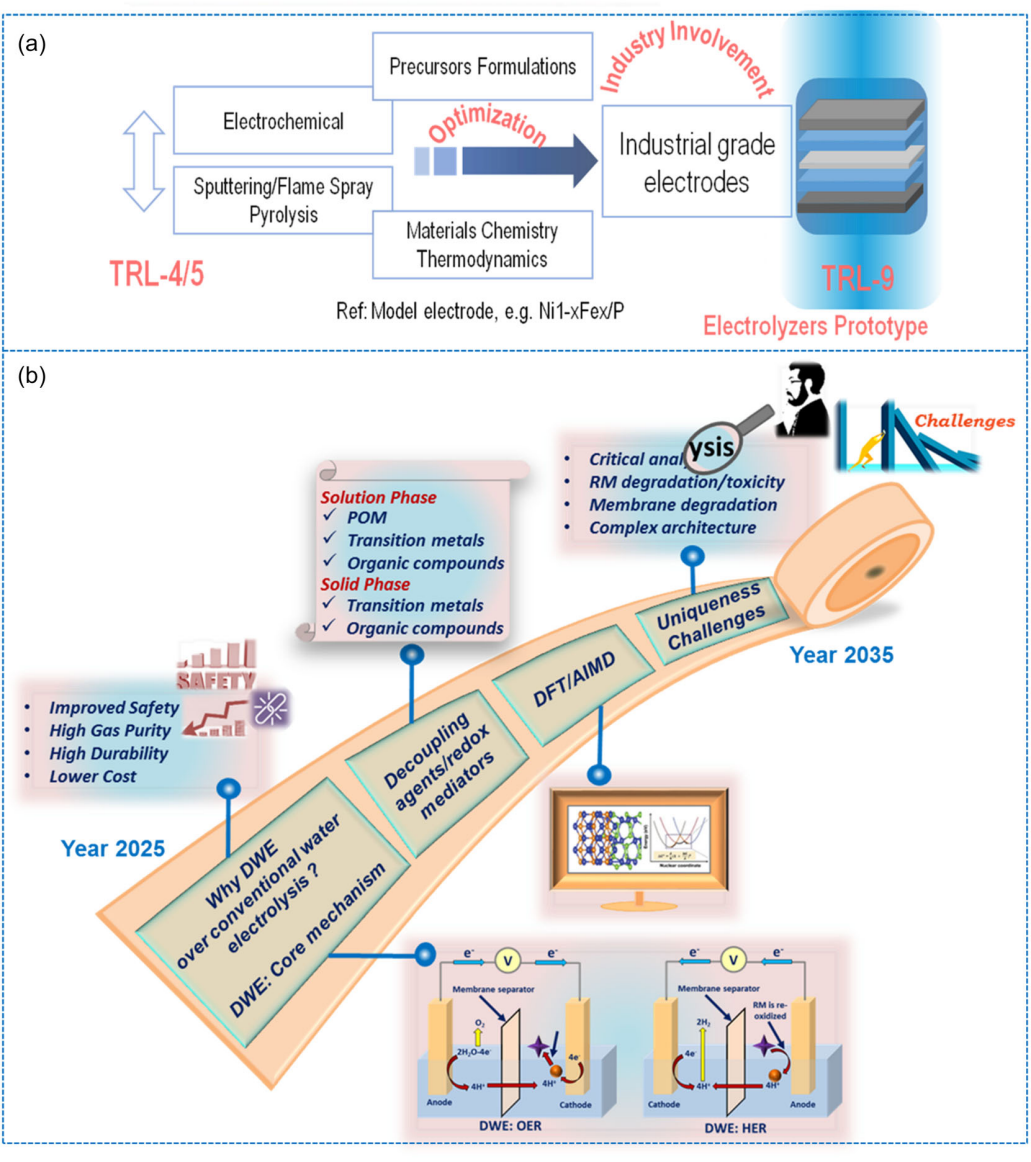
Industrial roadmap for decoupled water electrolysis (DWE). (a) Progression from TRL 4/5 to TRL 9, illustrating the translation of laboratory‐scale electrode technologies into industrial‐grade electrolyzer prototypes through materials optimization and scalable manufacturing. (b) Future development roadmap (2025–2035) highlighting key opportunities, research directions, and technological challenges for the commercialization of DWE systems.

## 11 Novelty and Scope of the Review

The previous reviews have highlighted the individual aspects of DWE technology, including redox mediators, decoupling mechanisms, membrane and membraneless architectures, catalyst development, renewable‐energy integration, and commercialization challenges. The present review therefore does not claim novelty simply from covering these established topics; rather, its scope is to integrate these previously reported aspects into a unified framework for evaluating DWE technologies. The key contribution of this review is the systematic analysis of performance parameters, such as current density, cell voltage, FE, gas purity/crossover, durability, scalability, safety, cost, and technology readiness of the DWE technology. Additionally, the review explains an integrated multiscale computational perspective on material design or architecture design, with the help of DFT, AIMD, continuum modeling, and machine‐learning approaches, which can help in the selection and optimization of redox mediators, electrocatalysts, and device architectures. Thus, the topics already extensively demonstrated in the previous literature are presented primarily as the necessary foundation in the present review. Moreover, greater priority is given to unified performance benchmarking, quantitative cross‐system analysis, and computation‐guided structure–property–performance relationships. Additionally, the review connects these analyses with techno‐economic considerations and technology readiness to identify the key material, operational, and system‐level barriers that must be addressed for DWE to progress from laboratory demonstrations toward industrial green hydrogen production. This integrated perspective is intended to distinguish the present review from earlier specialized reviews and provides a consistent framework for assessing the current status and future development of DWE technologies by emphasizing the mediator chemistry, membrane technologies, catalyst families, reactor configurations, techno‐economic analysis, TRL assessment, and computational insights as interconnected elements of its scope.

## 12 Conclusions

DWE offers an alternative innovative technique for green hydrogen production, which is much safer and flexible than conventional electrolysis. DWE separates the OER and HER either in time or space, thus enables to overcome the major drawbacks originated from conventional electrolyzers such as gas cross‐over at low potential, safety issues related to system pressure, and very poor compatibility with intermittent power supply. Therefore, integration with redox mediators and advanced modular architecture can make the DWE for wide distribution of green hydrogen. The most important features such as the cost‐effectiveness, rigid and highly stable redox mediators, improved electrode stability, tunable mediator conductivity, as well as the simple architecture make the DWE a superior green hydrogen production technology to the traditional technologies. However, as the DWE technology is new in the energy refore, some standard operating protocols are required to accelerate its pathway for technology transfer. The DWE technology can hold promises to excel the global energy transition for next decade. The technology is expected to evolve from laboratory‐scale prototypes to commercial systems with cost‐effective redox mediators, optimized cell designs, and scalable manufacturing processes. The adaptability with renewable energy infrastructure will significantly improve its economic viability and operational flexibility. The in silico study can accelerate the development of novel mediators and electrocatalysts, as well as it can help in new process development. By addressing the technological challenges and adopting the futuristic roadmap, DWE technology can be a well‐defined solution for sustainable clean energy production and successful milestone for decarbonizing the ecosystem and broader renewable energy transition.

## Author Contributions

The manuscript was drafted by A.C. in close discussions with J.D. and U.P. The review concept was conceived and developed by J.D. and U.P., while B.M.A. contributed in writing the interlinked DFT/AIMD observations and studies.

## Funding

This work was supported by the National Institute of Ocean Technology, Ministry of Earth Sciences (GAP‐1086) and DST, Government of India (DST/WISESCOPE/EWWM/2024/38).

## Conflicts of Interest

All authors hereby declare no known competing financial interests or personal relationships that could influence the consideration or evaluation of the work reported in this paper. J.D. is inventor of a Swedish patent on 7 December 2021 (no. SE2151488‐0) was approved as SE 546000 (2024/4/9) and PCT patent application (no PCT/ EP2022/084778) was filed on 7 December 2022, leading to US patent application US20250027212A1, held by CAPLYZER AB, where J.D. is a major shareholder. The other authors declare that they have no competing interests.

## Data Availability

Data sharing is not applicable to this article as no datasets were generated or analysed during the current study.
